# Morinagadepsin, a Depsipeptide from the Fungus *Morinagamyces vermicularis* gen. et comb. nov.

**DOI:** 10.3390/microorganisms9061191

**Published:** 2021-05-31

**Authors:** Karen Harms, Frank Surup, Marc Stadler, Alberto Miguel Stchigel, Yasmina Marin-Felix

**Affiliations:** 1 Department Microbial Drugs, Helmholtz Centre for Infection Research, Inhoffenstrasse 7, 38124 Braunschweig, Germany; Karen.Harms@helmholtz-hzi.de (K.H.); Marc.Stadler@helmholtz-hzi.de (M.S.); 2 Institute of Microbiology, Technische Universität Braunschweig, Spielmannstrasse 7, 38106 Braunschweig, Germany; 3 Mycology Unit, Medical School and IISPV, Universitat Rovira i Virgili, C/ Sant Llorenç 21, 43201 Reus, Tarragona, Spain; albertomiguel.stchigel@urv.cat

**Keywords:** cytotoxicity, depsipeptide, *Morinagamyces*, morinagadepsin, Schizotheciaceae, secondary metabolites, Sordariales

## Abstract

The new genus *Morinagamyces* is introduced herein to accommodate the fungus *Apiosordaria vermicularis* as inferred from a phylogenetic study based on sequences of the internal transcribed spacer region (ITS), the nuclear rDNA large subunit (LSU), and partial fragments of ribosomal polymerase II subunit 2 (*rpb*2) and β-tubulin (*tub*2) genes. *Morinagamyces vermicularis* was analyzed for the production of secondary metabolites, resulting in the isolation of a new depsipeptide named morinagadepsin (**1**), and the already known chaetone B (**3**). While the planar structure of **1** was elucidated by extensive 1D- and 2D-NMR analysis and high-resolution mass spectrometry, the absolute configuration of the building blocks Ala, Val, and Leu was determined as -l by Marfey’s method. The configuration of the 3-hydroxy-2-methyldecanyl unit was assigned as 22*R*,23*R* by *J*-based configuration analysis and Mosher’s method after partial hydrolysis of the morinagadepsin to the linear derivative compound **2**. Compound **1** showed cytotoxic activity against the mammalian cell lines KB3.1 and L929, but no antimicrobial activity against the fungi and bacteria tested was observed, while **2** was inactive. Compound **3** was weakly cytotoxic against the cell line L929, but did not show any antimicrobial activity.

## 1. Introduction

The genus *Apiosordaria* was introduced by von Arx and Gams in 1967 to accommodate *Pleurage verruculosa*, which differs from the other species of the genus by its ornamented ascospores [1]. *Apiosordaria* included species with two-celled ascospores with an ellipsoidal to subglobose ornamented upper cell, and with a triangular to cylindrical mostly smooth-walled lower cell [1,2,3]. The genera *Apiosordaria* and *Triangularia* were traditionally segregated by the shape of the upper cell of their ascospores, which are more or less conical in *Triangularia fide* Guarro and Cano [2]. However, in a recent phylogenetic study, the type strains of both genera were placed in the same monophyletic clade of the family Podosporaceae, resulting in the synonymization of *Apiosordaria* with *Triangularia* [4]. However, most species of *Apiosordaria* remained with an uncertain taxonomic placement. Subsequently, *A. sacchari* and *A. striatispora* were transferred to *Triangularia*, and *A. globosa*, *A. hispanica*, and *A. vestita* to *Jugulospora*, based on phylogenetic and morphological data [5]. In the present phylogenetic study, based on analysis of sequences of the internal transcribed spacer region (ITS), the nuclear rDNA large subunit (LSU), and fragments of ribosomal polymerase II subunit 2 (*rpb2*) and β-tubulin (*tub2*) genes, the new genus *Morinagamyces* is introduced to accommodate *A. vermicularis*, with phylogenetic affiliation to the recently established family Schizotheciaceae [5].

The order Sordariales includes producers of a great diversity of biologically active secondary metabolites [6,7,8,9], with potential uses as drugs. In this context, the ex-type strain of *Morinagamyces vermicularis* was tested for the production of bioactive compounds, leading to the isolation of a new depsipeptide named morinagadepsin (**1**), whose structure elucidation, antimicrobial activity, and cytotoxicity are presented herein. Chaetone B (**3**) was also produced, and its antimicrobial and cytotoxic activity tested.

## 2. Materials and Methods

### 2.1. Molecular Study

DNA of the ex-type strain of *Apiosordaria vermicularis* was extracted and purified directly from colonies according to the Fast DNA Kit protocol (MP Biomedicals, Solon, Ohio). The amplification of the ITS, D1−D3 domains of the LSU, *rpb2*, and *tub2* was performed according to White et al. [10] (ITS), Vilgalys and Hester [11] (LSU), and Miller and Huhndorf [12] (*rpb2* and *tub2*). PCR products were purified and sequenced at Macrogen Europe (Amsterdam, The Netherlands) with a 3730XL DNA analyzer (Applied Biosystems). Consensus sequences were obtained using SeqMan (version 7.0.0; DNASTAR, Madison, WI, USA). The phylogenetic analysis was carried out based on the combination of the four loci (ITS, LSU, *rpb2*, and *tub2*) sequences of the ex-type strain of *A. vermicularis* and selected members of the Sordariales, with *Camarops amorpha* SMH 1450 as outgroup. Each locus was aligned separately using MAFFT v. 7 [13] and manually adjusted in MEGA v. 10.2.4 [14]. Individual gene phylogenies were checked for conflicts before the four loci datasets were concatenated [15,16]. The Maximum Likelihood (ML) and Bayesian Inference (BI) analysis including the four loci were performed as described by Harms et al. [9]. The best evolutionary model for each sequence dataset was calculated using MrModeltest v. 2.3 [17]. Bootstrap support (bs) ≥ 70% and posterior probability values (pp) ≥ 0.95 were considered significant [18]. The sequences generated in this study are deposited in GenBank (Table 1) and the alignment used in the phylogenetic analysis is deposited in TreeBASE (S28234).

### 2.2. Fermentation and Extraction

The fungus was grown in yeast malt extract agar (YM agar; malt extract 10 g/L, yeast extract 4 g/L, d-glucose 4 g/L, agar 20 g/L, pH 6.3 before autoclaving [33]) at 23 °C. Later, the colonies were cut into small pieces using a cork borer (1 cm × 1 cm) and 8 pieces were placed into a 200 mL Erlenmeyer flask containing 100 mL of yeast malt extract broth (YM broth; malt extract 10 g/L, yeast extract 4 g/L, d-glucose 4 g/L, pH 6.3 before autoclaving) at 23 °C and under shake condition at 140 rpm. After 7 days, 6 mL of this seed culture were transferred to 40 conical flasks of 500 mL containing solid rice medium (BRFT, brown rice 28 g as well as 0.1 L of base liquid (yeast extract 1 g/L, di-sodium tartrate di-hydrate 0.5 g/L, KH_2_PO_4_ 0.5 g/L [34])) per flask. The rice cultures were incubated for 15 days at 23 °C.

For compound extraction, the mycelia in BRFT were covered with acetone, and sonicated in an ultrasonic bath for 30 min at 40 °C. The acetone extract was separated from the mycelium by filtration throughout a cellulose filter paper (MN 615 1/4 Ø 185 mm, Macherey-Nagel GmbH & Co. KG, Düren, Germany), and the mycelium was sonicated and extracted again. Both extracts were combined, and the acetone was evaporated to an aqueous residue in vacuo at 40 °C. The resulting aqueous phase was extracted twice with an equal amount of ethyl acetate in a separatory funnel. The ethyl acetate fraction was evaporated to dryness in vacuo (evaporator: Heidolph Instruments GmbH & Co. KG, Germany; pump: Vacuubrand GmbH & Co. KG, Wertheim am Main, Germany) at 40 °C. Afterwards, the ethyl acetate extract was dissolved in methanol. This was followed by extraction with an equal amount of heptane in a separatory funnel. This later step was repeated with the methanol phase obtained, which was evaporated to dryness in vacuo at 40 °C. The extracts were combined, dried in vacuo at 40 °C and weighed. Methanol extract yield was 1345 mg.

### 2.3. Compound Isolation

For compound isolation, the methanol extract dissolved in MeOH was portioned to 5 × 269 mg and separated using a PLC 2250 preparative HPLC system (Gilson, Middleton, WI, USA) with a Nucleodur^®^ C18ec column (125 × 40 mm, 7 µm; Macherey-Nagel, Düren, Germany) as stationary phase and the following conditions: solvent A: H_2_O + 0.1% formic acid, solvent B: Acetonitrile (ACN) + 0.1% formic acid; flow: 45 mL/min, fractionation: 15 mL, gradient: isocratic conditions at 20% B for 2 min, followed by an increase to 32% B in 8 min, then increase to 65% B in 25 min, followed by an increase to 100% B in 10 min, followed by isocratic conditions of 100% B for 10 min. This yielded compound **1** (48.4 mg, *t_R_* = 43.5–44 min) and compound **3** (6.2 mg, *t_R_* = 37–37.5 min).

### 2.4. Chromatography and Spectral Methods

Crude extract and pure compounds were dissolved to a concentration of 4.5 and 1 mg/mL, respectively, in an acetone and methanol solution (1:1). Then they were analyzed in an UltiMate^®^ 3000 Series UHPLC system (Thermo Fisher Scientific, Waltman, MA, USA) connected to an ion trap mass spectrometer (ESI-Ion Trap-MS, amazon speed, Bruker, Billerica, MA, USA), utilizing a C18 Acquity^®^ UPLC BEH column (2.1 × 50 mm, 1.7 m; Waters, Milford, MA, USA) to obtain the electrospray ionization mass spectra (ESI-MS). Solvent A was H_2_O + 0.1% formic acid and solvent B was ACN + 0.1% formic acid. The gradient started with 5% of solvent B for 0.5 min, followed by an increase to 100% B in 19.5 min, and maintained in 100% B for 5 min more, with a flow rate of 0.6 mL/min. The UV/vis spectra were recorded by diode array detection (DAD) in a range from 190–600 nm.

High-resolution electrospray ionization mass spectra (HR-ESI-MS) were recorded with an Agilent 1200 Infinity Series HPLC-UV system (Agilent Technologies, Santa Clara, CA, USA) connected to a time-of-flight mass spectrometer (ESI-TOF-MS, Maxis, Bruker, Billerica, MA, USA) (scan range 100–2500 *m*/*z*, rate 2 Hz, capillary voltage 4500 V, dry temperature 200 °C), using the same HPLC conditions described in ESI-MS measurements.

The 1D- and 2D- nuclear magnetic resonance (NMR) spectra of compounds **1** and **2** were recorded with an Avance III 700 spectrometer with a 5 mm TXI cryoprobe (Bruker, ^1^H NMR: 700 MHz, ^13^C: 175 MHz, Billerica, MA, USA) and an Avance III 500 (Bruker, ^1^H NMR: 500 MHz, ^13^C: 125 MHz, Billerica, MA, USA) spectrometer, respectively. The chemical shifts δ were referenced to the solvents DMSO-*d*_6_ (^1^H, δ = 2.50; ^13^C, δ = 39.51), and pyridine-*d*_5_ (^1^H, δ = 7.22; ^13^C, δ = 123.87).

Optical rotations were measured with an MCP 150 circular polarimeter at 20 °C (Anton Paar, Graz, Austria) and UV/Vis spectra with a UV-2450 spectrophotometer (Shimadzu, Kyoto, Japan). The optical rotation was obtained in MeOH and the UV/Vis spectra were measured in ACN.

### 2.5. Spectral Data

#### 2.5.1. Morinagadepsin (**1**)

White powder; [α]^20^_D_ −93° (c 0.001, MeOH); UV (ACN) λ_max_ (log ε) 194 (4.2); ^1^H-NMR and ^13^C-NMR see Table 2; ESI-MS: *m*/*z* 579 (M − H)^−^ and 581 (M + H)^+^; HR-ESI-MS: *m*/*z* 581.4275 (M + H)^+^ (calculated for C_31_H_57_N_4_O_6_, 581.4278).

#### 2.5.2. Chaetone B (**3**)

White to yellow oil; ^1^H-NMR and ^13^C-NMR were in good agreement with the literature [35]; ESI-MS: *m*/*z* 301 (M + H)^+^; HR-ESI-MS: *m*/*z* 301.1068 (M + H)^+^ (calculated for C_17_H_17_O_5_, 301.1076).

### 2.6. Determination of Amino Acid Stereochemistry

Determination of amino acid stereochemistry of **1** was performed with Marfey’s reagent (1-fluoro-2,4-dinitrophenyl-5-l-valinamide (FDAA) (Sigma-Aldrich, Deisenhofen, Germany)) following the protocol described by Viehrig et al. [36] with slight modifications. Compound **1** (1 mg) was hydrolyzed with 6 N HCl at 90 °C for 18 h. The hydrolysate was evaporated to dryness and redissolved in water (200 µL). Then, 1 N NaHCO_3_ (20 µL) and 1% FDAA (100 µL in acetone) were added. The solution was heated at 40 °C for 40 min. After cooling down, the solution was neutralized with 2 N HCl using pH paper and the sample was dried. The amino acids found in **1** were used as standards (d-l-Val (Sigma-Aldrich, Deisenhofen, Germany), l-Val (Sigma-Aldrich, Deisenhofen, Germany), d-l-Leu (Sigma-Aldrich, Deisenhofen, Germany), l-Leu (Sigma-Aldrich, Deisenhofen, Germany), l-Ala (Merck KGaA, Darmstadt, Germany), and d-Ala (Sigma-Aldrich, Deisenhofen, Germany)) and treated as explained above for the hydrolysate of **1**. All the resulting products were dissolved in 1 mL MeOH and analyzed with the UHPLC system connected to an ion trap mass spectrometer described above. The retention times in minutes of the FDAA-derivatized amino acids were Ala 5.1; Leu 7.4; and Val 6.5. Retention time of the FDAA-derivatized standards were l-Ala 5.0; d-Ala 5.8 *m*/*z* 340 (M - H)^−^; l-Leu 7.4; d-Leu 8.4 *m*/*z* 382 (M − H)^−^; l-Val 6.5; and d-Val 7.5 *m*/*z* 368 (M - H)^−^. The retention times showed that compound **1** is built with l-amino acids.

### 2.7. Partial Hydrolysis of Morinagadepsin to Compound ***2***

For the hydrolysis of **1**, the protocol described by Kwon et al. [37] was followed with slight modification. A portion of compound **1** (14.5 mg) was dissolved in 1 mL of 5% NaOMe (dissolved in methanol) and stirred for 20 h at 40 °C. Afterwards, the reaction was neutralized with 1 N HCl using pH paper and evaporated to dryness. Preparative HPLC used an Agilent 1100 series system (Santa Clara, CA, USA) with a Gemini^®^ C18ec column (250×21.2 mm, 7 µm; Phenomenex, Torrance, CA, USA) as stationary phase and the following conditions: solvent A: H_2_O + 0.1% formic acid, solvent B: ACN + 0.1% formic acid; flow: for 2 min at 17 to 20 mL/min and afterwards at 20 mL/min; fractionation: 10 mL/min; and gradient: isocratic conditions at 5% B for 2 min, followed by an increase to 55% B in 8 min, then increase to 65% B in 30 min, followed by an increase to 100% B in 10 min, followed by isocratic conditions of 100% B for 10 min. This yielded the pure compound **2** (7.9 mg, *t_R_* = 23.8–24.8 min).

#### 2.7.1. Spectral Data of the Linear Peptide (**2**)

White powder; [α]20D −30° (c 0.001, MeOH); UV (ACN) λmax (log ε) 192 (4.1); ^1^H-NMR δ_H_ 9.54 (br d, *J* = 8.4 Hz, 11-NH), 9.43 (br d, *J* = 7.3 Hz, 8-NH), 9.23 (br d, *J* = 5.6 Hz, 2-NH), 8.91 (br d, *J* = 9.0 Hz, 17-NH), 5.28–5.20 (m, 2-H, 8-H, 11-H), 5.09 (t, *J* = 8.3 Hz, 17-H), 4.09 (m, 23-H), 2.91 (qd, *J* = 7.1 Hz, 5.9 Hz, 22-H), 2.44 (dspt, *J* = 8.2 Hz, 6.9 Hz, 18-H), 2.06–1.82 (m, 3-H_2_, 4-H, 12-H_2_, 13-H), 1.79 (m, 24-H_2_), 1.73 (m, 25-H_a_), 1.61 (d, *J* = 7.1 Hz, 9-H_3_), 1.57 (m, 25-H_b_), 1.44 (d, *J* = 7.1 Hz, 31-H_3_), 1.29–1.18 (m. 26-H_2_ - 29-H_2_), 1.17 (d, *J* = 6.9 Hz, 20-H_3_), 1.00 (d, *J* = 6.5 Hz, 5-H_3_), 0.91 (d, *J* = 6.5 Hz, 6-H_3_), 0.84 (m, 14-H_3_), 0.83 (t, *J* = 6.9 Hz, 30-H_3_), 0.81 (d, *J* = 6.5 Hz, 15-H_3_); ESI-MS: *m*/*z* 597 (M − H)^−^ and 599 (M + H)^+^; HR-ESI-MS: *m*/*z* 599.4370 (M + H)^+^ (calculated for C_31_H_59_N_4_O_7_, 599.4384).

#### 2.7.2. Derivatization with MTPA

Compound **2** (1 mg) was dissolved in pyridine-*d*_5_ (0.6 mL), transferred into a NMR tube and then (*R*)- (−)-α-methoxy-α-(trifluoromethyl) phenylacetyl chloride (10 µL) was added. The mixture was incubated for 2 h at room temperature before the measurement of ^1^H, COSY, TOCSY, HSQC and HMBC NMR spectra was taken. This resulted in a (*S*)-MTPA ester derivative: ^1^H NMR (700 MHz, pyridine-*d*_5_): similar to **2**, but *δ*_H_ 9.05 (m, 17-NH), 5.90 (m, 23-H), 4.85 (m, 17-H), 3.30 (m, 22-H), 2.35 (m, 18-H), 2.01 (m, 24-H_a_), 1.78 (m, 24-H_b_), 1.47 (m, 25-H_2_), 1.27 (m, 31-H_3_), 1.27 (m, 26-H_2_), 1.21 (m, 29-H_2_), 1.20 (m, 27-H_2_), 1.18 (m, 28-H_2_), 1.14 (m, 19-H_3_), 1.03 (m, 20-H_3_), 0.83 (t, *J* = 7.3 Hz, 30-H_3_). The (*R*)-MTPA ester derivative was yielded analogously with (*S*)- (+)-α-methoxy-α-(trifluoromethyl) phenylacetyl chloride (10 µL). The reaction was incubated in pyridine-*d*_5_ (0.6 mL) for 65 h at room temperature: ^1^H NMR (700 MHz, pyridine-*d*_5_): similar to **2**, but *δ*_H_ 9.22 (m, 17-NH), 5.87 (m, 23-H), 4.90 (m, 17-H), 3.28 (m, 22-H), 2.42 (m, 18-H), 1.97 (m, 24-H_a_), 1.67 (m, 24-H_b_), 1.32 (m, 31-H_3_), 1.21 (m, 25-H_2_, 29-H_2_), 1.19 (m, 19-H_3_), 1.18 (m, 26-H_2_), 1.13 (m, 28-H_2_), 1.12 (m, 27-H_2_), 1.10 (m, 20-H_3_), 0.84 (m, 30-H_3_).

### 2.8. Antimicrobial and Cytotoxic Activity Assays

The antimicrobial activity was evaluated by determining the minimum inhibitory concentration (MIC) in 96-well round-bottom plates. Compounds **1, 2,** and **3** were tested against five fungi (i.e., *Candida albicans*, *Mucor hiemalis*, *Rhodotorula glutinis*, *Schizosaccharomyces pombe,* and *Wickerhamomyces anomalus*) and against bacteria (*Bacillus subtilis*, *Mycolicibacterium smegmatis,* and *Staphylococcus aureus* (Gram-positive), as well as *Acinetobacter baumanii*, *Chromobacterium violaceum*, *Escherichia coli*, and *Pseudomonas aeruginosa* (Gram-negative)). The cell suspension of most bacteria was done in Mueller–Hinton Broth (SN X927.1, Carl Roth GmbH, Karlsruhe, Germany) and was adjusted at OD_600_ nm to 0.01. *Mycolicibacterium smegmatis* was cultured in 27H9 + ADC (Middlebrook 7H9 Broth Base + Middlebrook ADC Growth Supplement (SN M0678 + M0553, Merck, Darmstadt, Germany)) and adjusted at OD_548_ nm to 0.1. The fungi were grown in MYC (1% bacto peptone, 1% yeast extract, 2% glycerol, pH 6.3) and adjusted at OD_548_ nm to 0.1. Then, 150 µL of the adjusted suspension was added to all wells of a 96-well microtiter plate. In row A, an additional 130 µL of suspensions plus 20 µL of the test compounds (1 mg/mL in methanol) were added. MeOH (20 µL) was used as negative control, while different positive controls were used depending on the test organisms. Nystatin (1 mg/mL) was used as positive control against the fungi. Oxytetracycline (0.1 mg/mL, *B subtilis* 1 mg/mL) was used for the bacteria, except for *Ac. baumanii*, *M. smegmatis*, and *P. aeruginosa*, against which cibrobay (0.25 mg/mL), kanamycin (0.1 mg/mL), and gentamycin (0.1 mg/mL) were used, respectively. Then, starting from row A, 150 µL of the suspension were transferred to the next row, and 150 µL transferred to the following row. The remaining 150 µL after row H were discarded. This resulted in a serial dilution of the test compounds, ranging from 66.7 µg/mL in row A to 0.52 µg/mL in row H. The assay was incubated overnight at 800 rpm on a microplate shaker. The temperature was chosen due to the microorganisms. They were grown at 30 °C, except *M. smegmatis*, *E. coli*, and *P.* aeruginosa which were grown at 37 °C. The lowest concentration of the compounds preventing visible growth of the test organism was recorded as the MIC.

The cytotoxicity of compounds **1, 2,** and **3** were tested against the two mammalian cell lines KB 3.1 (human endo-cervical adenocarcinoma) and L929 (mouse fibroblasts) in a 96-well plate. The compounds were dissolved as described in the previous section and epothilone B was used as the positive control. The cell lines were incubated with a serial dilution assay of the compounds (final range: 37 to 0.6 × 10^−3^ µg/mL) at 37 °C with 10% CO_2_ in Gibco™ Dulbecco’s Modified Eagle Medium (SN 61965026, Thermo Fisher Scientific, Waltham, MA, USA) supplemented with 10% Fetal Bovine Serum (SN 10500064, Thermo Fisher Scientific). After five days the cells were stained with 3-(4,5-dimethyl-2-thiazolyl)-2,5-diphenyl-2H-tetrazolium bromide (MTT, (M2128, Sigma-Aldrich, Deisenhofen, Germany)). The dye is converted to its purple derivative by living cells. The intensity of the purple derivative in the relation to the cells without additive (100% viability) was quantified for each concentration of the test compound. For the quantification, a microplate reader with 595 nm was used to calculate the percentage of the cell viability. From these results, the half-maximum inhibitory concentration (IC_50_) in µM was calculated.

## 3. Results

### 3.1. Molecular Phylogeny and Taxonomy

**Figure 1:**
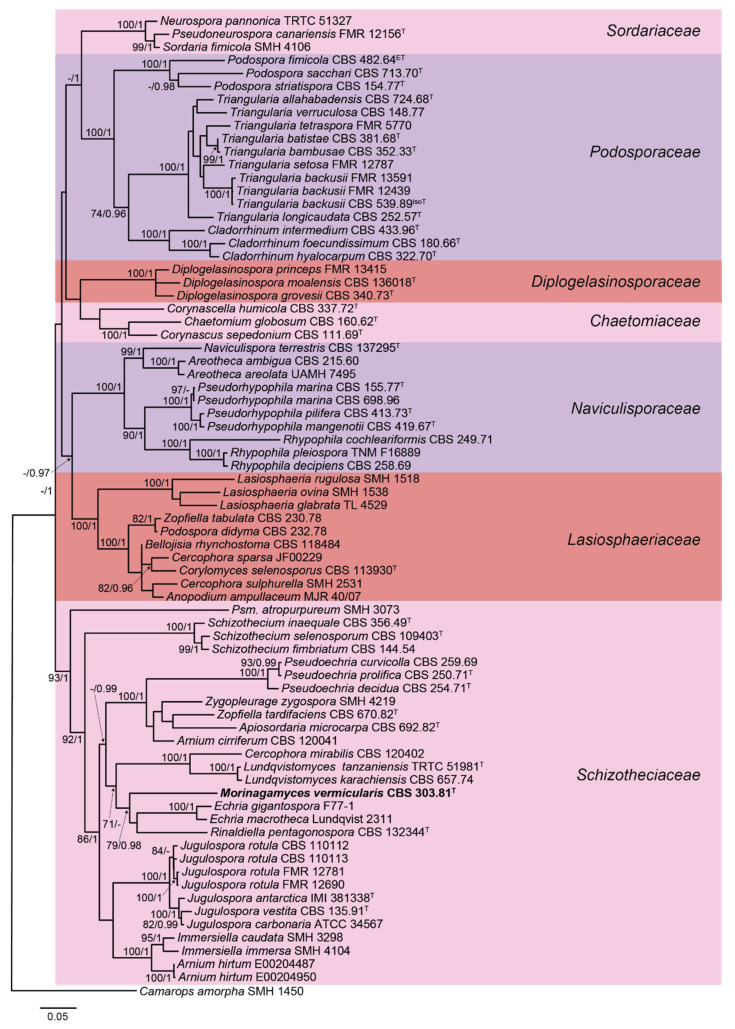
RAxML phylogram obtained from the combined sequences of the internal transcribed spacer region (ITS), the nuclear rDNA large subunit (LSU), and fragments of ribosomal polymerase II subunit 2 (*rpb2*) and β-tubulin (*tub2*) genes of selected strains belonging to the families Chaetomiaceae, Diplogelasinosporaceae, Lasiosphaeriaceae, Naviculisporaceae, Podosporaceae, Schizotheciaceae, and Sordariaceae. *Camarops amorpha* was used as outgroup. Bootstrap support values ≥ 70/Bayesian posterior probability scores ≥0.95 are indicated along branches. Branch lengths are proportional to distance. Novelty is indicated in **bold**. Ex-epitype, ex-isotype, and ex-type strains of the different species are indicated with ^ET^, ^IsoT^ and ^T^, respectively.

The lengths of the individual alignments used in the combined dataset were 681 bp (ITS), 894 bp (LSU), 984 bp (*rpb2*), and 618 bp (*tub2*), being the final total alignment of 3177 bp. The Maximum Likelihood tree obtained from the RAxML analysis of the combined dataset, including RAxML bootstrap support (BS) and Bayesian posterior probability at the nodes, is shown in Figure 1. For the BI analysis, the GTR + I + G model was selected for all partitions. The RAxML tree topology agreed with the topology of the tree generated by the BI analysis. The combined phylogenetic tree (Figure 1) showed seven main clades representing the families Chaetomiaceae, Diplogelasinosporaceae, Lasiosphaeriaceae, Naviculisporaceae, Podosporaceae, Schizotheciaceae, and Sordariaceae. The ex-type strain of *Apiosordaria vermicularis* was located in the family Schizotheciaceae, far from the *Triangularia* clade in Podosporaceae, where the type species of *Apiosordaria* (now *T. verruculosa*) is placed. *Apiosordaria vermicularis* formed a well-supported clade (79% bs/0.98 pp) with *Echria* spp. and *Rinaldiella pentagonospora*, but showed enough phylogenetic distance to propose it as the type species of the new genus *Morinagamyces*, as *Morinagamyces vermicularis*.

***Morinagamyces* Y. Marín and Stchigel, gen. nov.** MycoBank MB839453.

*Type species: Morinagamyces vermicularis* (Morinaga, Minoura and Udagawa) Y. Marín and Stchigel.

*Etymology*: Named in honor of the mycologist Tsutomu Morinaga, who collected and isolated the ex-type strain and introduced the basionym.

Ascomata non-ostiolate, scattered, semi-immersed to immersed, brownish black to black, opaque, globose to subglobose, glabrous or covered on upper exposed part with long, straight or flexuous, brown, septate, unbranched or branched, smooth-walled, hypha-like hairs; ascomatal wall brown to dark brown, membranaceous to coriaceous, three-layered; outer layer of textura intricata; middle layer composed of thin-walled, yellow brown to brown angular cells; inner layer composed of hyaline, flattened cells. Paraphyses filiform to ventricose, hyaline. Asci unitunicate, eight-spored, long cylindrical, often curved or sinuous, disposed in a basal fascicle, rounded apex, apical ring indistinct in the apex, non-amyloid, long-stipitate. Ascospores uniseriate, at first one-celled, hyaline, cylindrical-ellipsoidal, later becoming transversely uniseptate; upper cells dark olivaceous brown to dark brown, ovate to broadly ellipsoidal, truncate at base, rounded or slightly acuminate at apex, with walls ornamented by numerous, stiff warts, with a germ pore in apex; lower cell hyaline to pale brown, cylindrical to long triangular, frequently 1-septate, smooth-walled. Asexual morph of two types: (**1**) Conidiophores indistinguishable from the hyphae. Conidiogenous cells phialidic, integrated to hyphae, cylindrical, with a terminal collarette. Conidia hyaline, subglobose to ovate, smooth-walled, gathering in a globose, slimy mass; (**2**) Conidia holoblastic, borne along the sides of hyphae, sessile or short-stipitate, hyaline, pyriform to clavate, truncate at base, rounded apex, smooth-walled.

Notes: *Echinopodospora vermicularis* was introduced by Morinaga et al. to accommodate a fungus from soil in Hong Kong characterized by the production of non-ostiolate ascomata and ascospores with a warted upper cell [38]. Subsequently, it was transferred to *Apiosordaria*, when the genus *Echinopodospora* was synonymized with *Apiosordaria* based on their morphological similarities [39]. However, the phylogenetic data demonstrated that this species represents a new lineage in the recently introduced family Schizotheciaceae, and consequently the genus *Morinagamyces* was introduced. The main distinctive feature of this new genus is the presence of two different kinds of asexual morphs, i.e., cladorrhinum- and chrysosporium-like. This particular feature has only been reported in another species of the Sordariales, *A. effusa* [38], which has never been studied with molecular data, and its taxonomic position remains unresolved. Surprisingly, the cladorrhinum-like asexual morph is distinctive of the three genera belonging to the family Podosporaceae, i.e., *Cladorrhinum*, *Podospora*, and *Triangularia*, as it is not observed in any other families of the Sordariales.

The closest related genera to *Morinagamyces* are *Echria* and *Rinaldiella*. However, the later genera have not been reported to produce asexual morphs and they are characterized by the production of ostiolate ascomata, while *Morynagamyces* produces non-ostiolate ones. *Echria* can be easily distinguished from the other two genera by production of one-celled roughened or smooth-walled ascospores (two-celled and warted in the other two genera) [19]. *Morinagamyces* and *Rinaldiella* produce two-celled warted ascospores, but the upper cell is five-angled in side view in *Rinaldiella* [27], while it is ovate to broadly ellipsoidal in *Morinagamyces*.

***Morinagamyces vermicularis* (Morinaga, Minoura and Udagawa) Y. Marín and Stchigel, comb. nov.** MycoBank MB839454.

*Basionym: Echinopodospora vermicularis* Morinaga, Minoura and Udagawa, Trans. Mycol. Soc. Japan 19: 138. 1978.

*Synonym: Apiosordaria vermicularis* (Morinaga, Minoura and Udagawa) J.C. Krug, Udagawa and Jeng, Mycotaxon 17: 547. 1983.

### 3.2. Isolation and Structure Elucidation of Secondary Metabolites

**Figure 2:**
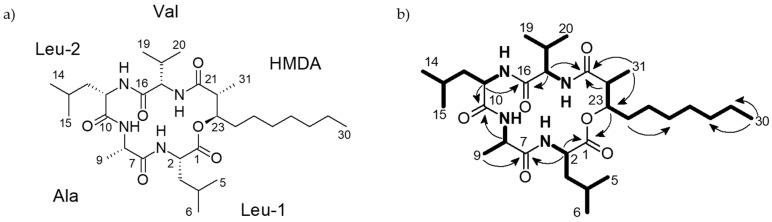
(**a**) Structure of morinagadepsin **1**. (**b**) selected ^1^H,^1^H COSY (bold lines) and ^1^H,^13^C HMBC (black arrows) correlations of **1**.

Morinagadepsin (**1**) was isolated as a white powder. Its molecular formula of C_31_H_56_N_4_O_6_ was derived from its HR-ESI-MS peak observed at *m*/*z* 581.4271, implying six degrees of unsaturation. ^1^H and HSQC (Heteronuclear single-quantum correlation spectroscopy) NMR spectra measured in DMSO-*d*_6_ specified the presence of nine methyls, seven methylenes, and nine methines, of which four were bound to nitrogen and one bound to oxygen, in addition to four exchangeable protons bound to heteroatoms. The ^13^C-NMR spectrum indicated the presence of five carbonyls. By COSY (correlation spectroscopy), TOCSY (total correlation spectroscopy) and intra-residue HMBC (heteronuclear multiple-bond correlation spectroscopy) correlations, alanine (Ala), valine (Val), and two leucine (Leu-1 and Leu-2), as well as 3-hydroxy-2-methyldecanoic acid (HMDA) residues were assembled (see Figure 2b). The sequence of entities was assigned by inter-residue ^1^H,^13^C HMBC correlations (Figure 2b). The low field shift of 23H (*δ*_H_ 4.92) indicated an ester linkage at this position, which was confirmed by the ^1^H,^13^C HMBC correlation of 23-H to C-1, establishing the planar depsipeptidal structure of **1**.

**Figure 3:**
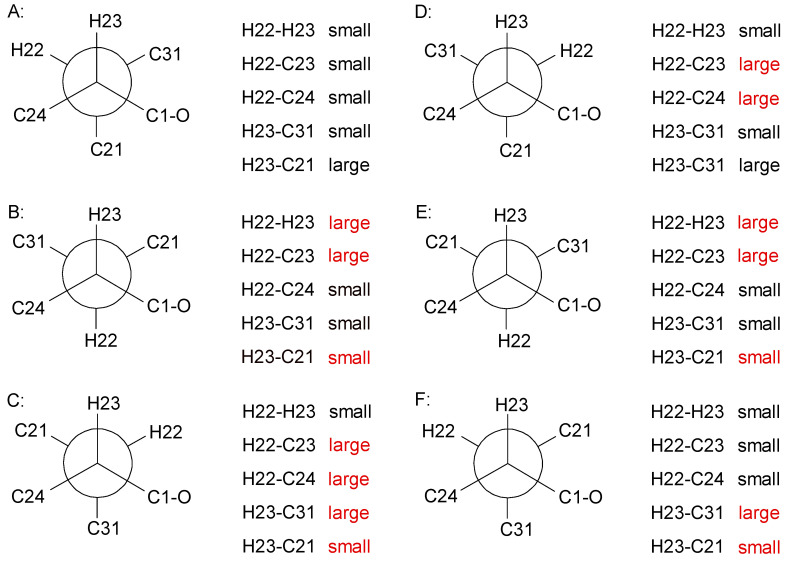
J-based analysis of six hypothetical rotamers with 22S,23S (**A**–**C**) and 22S,23R (**D**–**F**) configuration to determine the stereochemistry of **1**. Expected couplings contrary (shown in red) to the observed ones (^3^J(H22,H23) = 4.8 Hz; ^2^J(H22,C23) = 2.2 Hz; ^3^J(H22,C24) = 1.0 Hz; ^2^J(H23,C31) = 2.6 Hz; ^3^J(H23,C21) = 6.6 Hz) exclude all configurations except A. This rotamer is confirmed by ROESY correlations between 23-H/22-H, 23-H/31-H_3_, 24-H_a/b_/22-H, and 31-H_3_/3-H_b_.

After complete hydrolysis and derivatization with FDAA, we observed l-Leu, l-Val, and l-Ala according to Marfey’s method [36]. Thus, C-2, C-8, C-11, and C-17 are *S*-configurated. The relative configurations of the chiral centers C-22/C-23 in HMDA was determined by *J*-based configurational analysis using ^3^*J*_HH_, ^2^*J*_CH_, ^3^*J*_CH_ and ROESY correlations (Figure 3) as 22R*,23R*. Necessary proton-carbon coupling constants were obtained from a HSQC-Hecade NMR spectrum (Figure S10), except ^3^J(H23,C21) = 6.6 Hz, which was extracted from a *J*-HMBC NMR experiment (Figure S11) [40].

**Figure 4:**
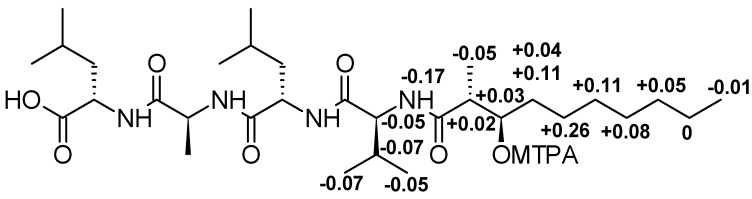
Δδ^SR^vvalues in ppm for the C-23-MTPA esters of **2** in pyridin-d_5_.

Finally, the absolute configuration of the HMDA subunit was determined by the derivatization of the methanolysis product with *S*- and *R*-MTPA according to Mosher’s method. The pattern of Δδ^SR^ values (see Figure 4) with negative values for 17-NH, 17-H, 18-H, 19-H_3_, 20-H_3_, and 31-H_3_, and positive ones for 24-H_2_ to 29-H_2_, is diagnostic for a 23*R* configuration [41].

### 3.3. Biological Activities

Compounds **1** and **2** were not active against the microorganisms tested in the serial dilution assay. Compound **3** showed weak activity against *B. subtilis* and *Mu. hiemalis* (Table 3).

Only compound **1** showed weak cytotoxic activity against the mammalian cell lines tested, while compound **3** was only weakly cytotoxic against the L929 cell line, and compound **2** did not have any activity (Table 4).

## 4. Discussion

The genus *Apiosordaria*, as well as other lasiosphaeriaceous genera, such as *Cercophora*, *Podospora*, and *Zopfiella*, resulted in a polyphyletic clade. Encompassing species scattered among the order Sordariales [4,5,12,20,28,32]. The main problem is that the traditional classification of the lasiosphaeriaceous taxa was based predominantly on the ascospore morphology, but this was found to be an extremely homoplastic character [12]. *Apiosordaria* was recently synonymized with *Triangularia*, located in the Podosporaceae, as the type species *A. verruculosa* formed a monophyletic clade with the type species of this later genus, *T. bambusae*, being consequently proposed the new combination *T. backusii* and *T. verruculosa* [4]. Subsequently, *A. sacchari* and *A. striatispora* were also transferred to *Triangularia*, whereas *A. antarctica*, *A. globosa*, *A. hispanica*, and *A. vestita* have been transferred to *Jugulospora*, phylogenetically located in the Schizotheciaceae [5]. However, a high number of *Apiosordaria* spp. remain in an uncertain taxonomic placement, as in the case of *A. microcarpa* (see Figure 1). In order to get a more natural classification of these species, the new genus *Morinagamyces* is introduced to accommodate *A. vermicularis*, which has proven to be an independent lineage in the Schizotheciaceae. This genus differs from the other taxa of the family, and even the order Sordariales, by the production of two types of asexual morphs, i.e., cladorrhinum- and chrysosporium-like. This characteristic was also observed in *A. effusa* [38]. Therefore, further studies will be needed to verify if this species also belongs to the genus *Morinagamyces*.

Morinagadepsin (**1**) belongs to the large class of depsipeptides, compounds containing both amide and ester bonds, which are widely distributed in nature. They have been isolated from plants, sponges and lower animals, cyanobacteria, bacteria, and fungi, with bioactivities ranging from antimicrobial, nematicidal, antiviral, and cytotoxic/cytostatic, to immunosuppressive and other pharmacologically important properties [42]. Fungal depsipeptides have been reported from many fungal genera, and it would lead too far to mention them all here. Some prominent examples are shown in Figure S23 (Supplementary Information). The nematicidal cyclodepsipeptide PF1022-A (Figure S23d), which has given rise to the marketed antiparasitic agent emodepside, had originally been discovered from an endophytic fungus associated with the tea plant, and only recently the affinities of the producer strain to the genus *Rosellinia* (Xylariaceae) were established [43]. The related cyclic hexadepsipeptide beauvericin (Figure S23a) probably acts as pathogenicity factor in the insect pathogenic *Beauveria* and *Isaria* species, and was also found in the genus *Fusarium* [44,45]. Verlamelin (Figure S23g) is another known depsipeptide with antifungal properties, produced by *Simplicillium* *lamellicola* (syn. V*erticillium lamellicola*) [46] and was eventually under development as antimycotic. Morinagadepsin belongs to the subgroup of cyclic pentadepsipeptides, which have been isolated from the genera *Acremonium, Alternaria, Fusarium, Hapsidospora,* and *Penicillium* [47]. Its hallmark is the presence of a 3-hydroxy-2-methyldecanoic acid (HMDA) moiety. HMDA has previously been detected as part of emericellamides C and D (Figure S23b) produced by *Aspergillus nidulans* [48], the lipopeptaibol trichopolyn V (Figure S23f) from *Trichoderma polysporum* [49], hapalosin (Figure S23c) from the cyanobacterium *Hapalasiphon welwitschii* [50], and the globomycin derivative SF-1902 A_4a_ (Figure S23e) from the bacterium *Streptomyces hygroscopicus* [51]. In the case of globomycin and its derivatives, the β-hydroxy-α-methyl carboxylic acid greatly contributes to the antibacterial activity [52]. Compound **1** did not show any activity against any microorganisms tested in the present study, but it was weakly cytotoxic against the two cell lines tested, while compound **2**, which is the linear peptide obtained from the partial hydrolysis of **1**, did not show antimicrobial or cytotoxic activity.

The other compound isolated from *M. vermicularis* was chaetone B (**3**). This compound was previously isolated from a strain of *Chaetomium* isolated from submerged woody substrate in fresh water [35], which is another member of the order Sordariales. Shen et al. [35] observed moderate activity of this compound against *S. aureus* in a standard disk assay. However, this compound did not show activity against this bacterium in our serial dilution assay. It showed weak bioactivity against the Gram-positive bacterium *B. subtilis*, and the fungus *Mu. hiemalis*. Compound **3** was also weakly cytotoxic against the L929 cell line.

## Figures and Tables

**Table 1:** Strains of the order Sordariales included in the phylogenetic study. GenBank accession numbers in **bold** correspond to the newly generated sequences. Taxonomic novelty is indicated in ***italic bold***.

Taxa	Strain	GenBank Accession Number				Source
		LSU	ITS	*rpb2*	*tub2*	
Anopodium ampullaceum*	MJR 40/07	KF557662	-	-	KF557701	[19]
Apiosordaria microcarpa*	CBS 692.82^T^	MK926841	MK926841	MK876803	-	[4]
*Areotheca ambigua*	CBS 215.60	AY999114	AY999137	-	-	[20]
*Areotheca areolata*	UAMH 7495	AY587936	AY587911	AY600275	AY600252	[21]
Arnium cirriferum*	CBS 120041	KF557673	-	-	KF557709	[19]
Arnium hirtum*	E00204950	KF557675	-	-	KF557711	[19]
	E00204487	KF557676	-	-	KF557712	[19]
Bellojisia rhynchostoma*	CBS 118484	EU999217	-	-	-	[22]
*Camarops amorpha*	SMH 1450	AY780054	-	AY780156	AY780093	[12]
*Cercophora mirabilis*	CBS 120402	KP981429	MT784128	KP981611	KP981556	[5]
Cercophora sparsa*	JF 00229	AY587937	AY587912	-	AY600253	[21]
Cercophora sulphurella*	SMH 2531	AY587938	AY587913	AY600276	AY600254	[21]
*Chaetomium globosum*	CBS 160.62^T^	MH869713	KT214565	KT214666	-	[23,24]
*Cladorrhinum foecundissimum*	CBS 180.66^T^	MK926856	MK926856	MK876818	-	[4]
*Cladorrhinum hyalocarpum*	CBS 322.70^T^	MK926857	MK926857	MK876819	-	[4]
*Cladorrhinum intermedium*	CBS 433.96^T^	MK926859	MK926859	MK876821	-	[4]
Corylomyces selenosporus*	CBS 113930^T^	DQ327607	MT784130	KP981612	KP981557	[5,25]
*Corynascus sepedonium*	CBS 111.69^T^	MH871003	MH859271	FJ666394	-	[23,26]
*Corynascella humicola*	CBS 337.72^T^	MH872209	MH860493	-	-	[23]
*Diplogelasinospora grovesii*	CBS 340.73^T^	MH872401	MH860693	-	-	[23]
*Diplogelasinospora moalensis*	CBS 136018^T^	KP981430	HG514152	KP981613	KP981558	[5,27]
*Diplogelasinospora princeps*	FMR 13415	KP981432	-	KP981615	KP981560	[5]
*Echria gigantospora*	F77-1	KF557674	-	-	KF557710	[19]
*Echria macrotheca*	Lundqvist 2311	KF557684	-	-	KF557715	[19]
*Immersiella caudata*	SMH 3298	AY436407	-	AY780161	AY780101	[12,28]
*Immersiella immersa*	SMH 4104	AY436409	-	AY780181	AY780123	[12,28]
*Jugulospora antarctica*	IMI 381338^T^	KP981433	-	KP981616	KP981561	[5]
*Jugulospora carbonaria*	ATCC 34567	AY346302	-	AY780196	AY780141	[12,29]
*Jugulospora rotula*	CBS 110112	KP981434	-	KP981617	KP981562	[5]
	CBS 110113	KP981435	-	KP981618	KP981563	[5]
	FMR 12690	KP981437	MT784133	KP981620	KP981565	[5]
	FMR 12781	KP981438	MT784134	KP981621	KP981566	[5]
*Jugulospora vestita*	CBS 135.91^T^	MT785872	MT784135	MT783824	MT783825	[5]
*Lasiosphaeria glabrata*	TL 4529	AY436410	AY587914	AY600277	AY600255	[21,28]
*Lasiosphaeria ovina*	SMH 1538	AF064643	AY587926	AY600287	AF466046	[21,30,31]
*Lasiosphaeria rugulosa*	SMH 1518	AY436414	AY587933	AY600294	AY600272	[21,28]
*Lundqvistomyces karachiensis*	CBS 657.74	KP981447	MK926850	KP981630	KP981478	[4,5]
*Lundqvistomyces tanzaniensis*	TRTC 51981^T^	AY780081	MH862260	AY780197	AY780143	[12,23]
***Morinagamyces vermicularis***	CBS 303.81^T^	**KP981427**	**MT904879**	**KP981609**	**KP981554**	Present study
*Naviculispora terrestris*	CBS 137295^T^	KP981439	MT784136	KP981622	KP981567	[5]
*Neurospora pannoica*	TRTC 51327	AY780070	-	AY780185	AY780126	[12]
Podospora didyma*	CBS 232.78	AY999100	AY999127	-	-	[20]
*Podospora fimicola*	CBS 482.64^ET^	KP981440	MK926862	KP981623	KP981568	[4,5]
*Podospora sacchari*	CBS 713.70^T^	KP981425	MH859915	KP981607	KP981552	[5,23]
*Podospora striatispora*	CBS 154.77^T^	KP981426	MT784137	KP981608	KP981553	[5]
*Pseudoechria curvicolla*	CBS 259.69	MH871036	MH859302	-	-	[23]
*Pseudoechria decidua*	CBS 254.71^T^	MK926842	MK926842	MK876804	-	[4]
*Pseudoechria. prolifica*	CBS 250.71^T^	MK926848	MK926848	MK876810	-	[5]
*Pseudoneurospora canariensis*	FMR 12156^T^	MH877580	-	-	HG423208	[23,27]
*Pseudorhypophila mangenotii*	CBS 419.67^T^	KP981444	MT784143	KP981627	KP981571	[5]
*Pseudorhypophila marina*	CBS 155.77^T^	MK926851	MK926851	MK876813	-	[4]
	CBS 698.96^T^	MK926853	MK926853	MK876815	-	[4]
*Pseudorhypophila pilifera*	CBS 413.73^T^	MK926852	MK926852	MK876814	-	[4]
*Pseudoschizothecium atropurpureum*	SMH 3073	AY780057	-	AY780160	AY780100	[12]
*Rinaldiella pentagonospora*	CBS 132344^T^	KP981442	MH866007	KP981625	KP981570	[5,23]
*Rhypophila cochleariformis*	CBS 249.71	AY999098	AY999123	-	-	[20]
*Rhypophila decipiens*	CBS 258.69	AY780073	KX171946	AY780187	AY780130	[12], Miller [unpubl. data]
*Rhypophila pleiospora*	TNM F16889	-	EF197084	-	-	[32]
*Schizothecium fimbriatum*	CBS 144.54	AY780075	AY999115	AY780189	AY780132	[12,20]
*Schizothecium inaequale*	CBS 356.49^T^	MK926846	MK926846	MK876808	-	[4]
*Schizothecium selenosporum*	CBS 109403^T^	MK926849	MK926849	MK876811	-	[4]
*Sordaria fimicola*	SMH 4106	AY780079	-	AY780194	AY780138	[12]
*Triangularia allahabadensis*	CBS 724.68^T^	MK926865	MK926865	MK876827	-	[4]
*Triangularia backusii*	CBS 539.89^IsoT^	MK926866	MK926866	MK876828	-	[4]
*Triangularia backusii*	FMR 12439	KP981423	MT784138	KP981605	KP981550	[5]
*Triangularia backusii*	FMR 13591	KP981424	MT784139	KP981606	KP981551	[5]
*Triangularia bambusae*	CBS 352.33^T^	MK926868	MK926868	MK876830	-	[4]
*Triangularia batistae*	CBS 381.68^T^	KP981443	MT784140	KP981626	KP981577	[5]
*Triangularia longicaudata*	CBS 252.57^T^	MK926871	MK926871	MK876833	-	[4]
*Triangularia setosa*	FMR 12787	KP981441	MT784144	KP981624	KP981569	[5]
*Triangularia tetraspora*	FMR 5770	AY999130	AY999108	-	-	Cai et al. [unpubl. data]
*Triangularia verruculosa*	CBS 148.77	MK926874	MK926874	MK876836	-	[4]
*Zopfiella tabulata*	CBS 230.78	MK926854	MK926854	MK876816	-	[4]
Zopfiella tardifaciens*	CBS 670.82^T^	MK926855	MK926855	MK876817	-	[4]
*Zygopleurage zygospora*	SMH 4219	AY346306	-	-	AY780147	[12,29]

ATCC: American Type Culture Collection, Virginia, USA; CBS: Westerdijk Fungal Biodiversity Institute, Utrecht, the Netherlands; FMR: Facultat de Medicina, Reus, Spain; IMI: International Mycological Institute, CABI-Bioscience, Egham, UK; TNM: Herbarium of National Museum of Natural Science, Taiwan; TRTC: Royal Ontario Museum, Toronto, Canada; UAMH: UAMH Center for Global Microfungal Biodiversity, University of Toronto, Canada; JF, Lundqvist, MJR, SMH, and TL: personal collections of Jacques Fournier, Nils Lundqvist, Michael J. Richardson, Sabine M. Huhndorf, and Thomas Læssøe, respectively; and n/a: not available. ^ET, IsoT and T^ indicates ex-epitype, ex-isotype and ex-type strains, respectively. * Taxa with generic names applied in the broad sense (sensu lato), not necessarily reflecting molecular phylogenetic relationships.

**Table 2:** NMR data (^1^H 700 MHz, ^13^C 175 MHz) of morinagadepsin **1** in DMSO-*d*_6_.

Atom#	Atom#	C Shift	H Shift	Atom#	Atom#	C Shift	H Shift
Leu1	1	170.9, C		Val	16	172.4, C	
	2	51.1, CH	4.39, ddd (9.5,9.0,5.8)		17	58.0, CH	4.01, t (7.4)
	2NH		7.80, br d (7.5)		17NH		6.91, d (7.4)
	3	39.6, CH_2_	1.74, m		18	30.5, CH	1.83, m
	3		1.67, m		19	18.74, CH_3_	0.87, m*
	4	24.1, CH	1.67, m		20	18.77, CH_3_	0.87, m*
	5	22.5, CH_3_	0.91, d (6.5)	HMD	21	172.5, C	
	6	21.7, CH_3_	0.86, m*		22	45.8, CH	2.47, qd (7.3, 4.8)
Ala1	7	171.5, C			23	75.1, CH	4.92, ddd (8.7, 4.8, 4.0)
	8	49.8, CH	3.96, qd (7.5, 6.0)		24	32.1, CH_2_	1.44, m*
	8NH		7.98, d (6.0)		24		1.35, m*
	9	16.1, CH_3_	1.37, d		25	24.5, CH_2_	1.05, m*
Leu2	10	170.7, C			26	28.59, CH_2_	1.19, m*
	11	53.7, CH	3.53, m		27	28.61, CH_2_	1.19, m*
	11NH		9.30, d (7.0)		28	31.1, CH_2_	1.19, m*
	12	36.6, CH_2_	1.54, m*		29	22.1, CH_2_	1.24, m*
	12		1.99, m		30	13.9, CH_3_	0.84, t (7.1)
	13	24.7, CH	1.57, m*		31	14.8, CH_3_	1.05, d (7.3)
	14	23.6, CH_3_	0.91, d (6.5)				
	15	22.5, CH_3_	0.87, m*				

* Signals overlapping in the ^1^H-NMR spectrum.

**Table 3:** Minimum inhibitory concentration (MIC, µg/mL) of **1–3** against bacterial and fungal test organisms.

Test Organism	Strain Number	1	2	3	Positive Control
*Bacillus subtilis*	DSM 10	–	–	66.6	8.30 ^O^
*Mycolicibacterium smegmatis*	ATCC 700084	–	–	–	1.70 ^K^
*Staphylococcus aureus*	DSM 346	–	–	–	0.83 ^O^
*Acinetobacter baumanii*	DSM 30008	–	–	–	0.53 ^C^
*Chromobacterium violaceum*	DSM 30191	–	–	–	0.83 ^O^
*Escherichia coli*	DSM 1116	–	–	–	1.70 ^O^
*Pseudomonas aeruginosa*	DSM 19882	–	–	–	0.42 ^G^
*Mucor hiemalis*	DSM 2656	–	–	66.6	2.10 ^N^
*Candida albicans*	DSM 1665	–	–	–	4.20 ^N^
*Rhodotorula glutinis*	DSM 10134	–	–	–	1.00 ^N^
*Schizosaccharomyces pombe*	DSM 70572	–	–	–	4.20 ^N^
*Wickerhamomyces anomala*	DSM 6766	–	–	–	4.20 ^N^

ATCC: American Type Culture Collection, Manassas, VA, USA; DSM: Leibniz-Institut DSMZ—German Collection of Microorganisms and Cell Cultures GmbH, Braunschweig, Germany. ^C^ cibrobay, ^G^ gentamicin, ^K^ kanamycin, ^N^ nystatin, and ^O^ oxytetracycline. –: no inhibition observed under test conditions.

**Table 4:** Cytotoxicity of **1–3** against mammalian cell lines (half maximal inhibitory concentrations (IC_50_): μM).

Cell Line	Number ^1^	IC_50_ [µM]			
		1	2	3	Epothilone B*
HeLa KB 3.1	ACC 158	37.2	–	–	0.00003
Mouse fibroblast L929	ACC 2	13.7	–	34.9	0.00051

–: no inhibition observed under test conditions. ^1^ ACC: Leibniz-Institut DSMZ—German Collection of Microorganisms and Cell Cultures GmbH, Braunschweig, Germany. * positive control (1 mg/mL).

## Data Availability

The DNA sequences are deposited in GenBank (https://www.ncbi.nlm.nih.gov/genbank/) and all other relevant data are included in the Supplementary Information.

## Supplementary Materials

The following are available online at https://www.mdpi.com/article/10.3390/microorganisms9061191/s1, Figure S1: HPLC-ESI-MS spectrum of morinagadepsin (**1**) in positive and negative; Figure S2: HPLC-HR-ESI-MS spectrum of morinagadepsin (**1**) in positive mode; Figure S3: ^1^H NMR spectrum (700 MHz, DMSO-*d*_6_) of morinagadepsin (**1**); Figure S4: ^13^C NMR spectrum (175 MHz, DMSO-*d*_6_) of morinagadepsin (**1**); Figure S5: COSY NMR spectrum (700 MHz, DMSO-*d*_6_) of morinagadepsin (**1**); Figure S6: HSQC NMR spectrum (700 MHz, DMSO-*d*_6_) of morinagadepsin (**1**); Figure S7: HMBC NMR spectrum (700 MHz, DMSO-*d*_6_) of morinagadepsin (**1**); Figure S8: ROESY NMR spectrum (700 MHz, DMSO-*d*_6_) of morinagadepsin (**1**); Figure S9: J-res NMR spectrum (700 MHz, DMSO-*d*_6_) of morinagadepsin (**1**); Figure S10: Sections from the HSQC-Hecade NMR spectrum (700 MHz, DMSO-*d*_6_) of morinagadepsin (**1**); Figure S11: J-HMBC NMR spectrum (700 MHz, DMSO-*d*_6_) of morinagadepsin (**1**); Figure S12: HPLC-ESI-MS spectrum of **2** in positive and negative mode; Figure S13: HPLC-HR-ESI-MS spectrum of **2** in positive mode; Figure S14: ^1^H NMR spectrum (700 MHz, pyridine-*d*_5_) of **2**; Figure S15: ^13^C NMR spectrum (175 MHz, pyridine-*d*_5_) of **2**; Figure S16: HSQC NMR spectrum (700 MHz, pyridine-*d*_5_) of **2**; Figure S17: COSY NMR spectrum (700 MHz, pyridine-*d*_5_) of **2**; Figure S18: HMBC NMR spectrum (700 MHz, pyridine-*d*_5_) of **2**; Figure S19: HSQC NMR spectrum (700 MHz, pyridine-*d*_5_) of the S-MTPA-ester of **2**; Figure S20: HSQC NMR spectrum (700 MHz, pyridine-*d*_5_) of the R-MTPA-ester of **2**; Figure S21: HPLC-ESI-MS spectrum of chaetone B (**3**) in positive and negative mode; Figure S22: HPLC-HR-ESI-MS spectrum of chaetone B (**3**) in positive mode; and Figure S23: Chemical structures of some known cyclodepsipeptides.
