# Supplementary material for: Morinagadepsin, a Depsipeptide from the Fungus Morinagamyces vermicularis gen. et comb. nov
Source: Microorganisms. 2021 May 31;9(6):1191. doi: 10.3390/microorganisms9061191 (PMC8230337; doi:10.3390/microorganisms9061191)
Supplement: Supplementary file 1 [file microorganisms-09-01191-s001.zip › microorganisms-1214271-supplementary.pdf]

microorganisms

Supplementary Material

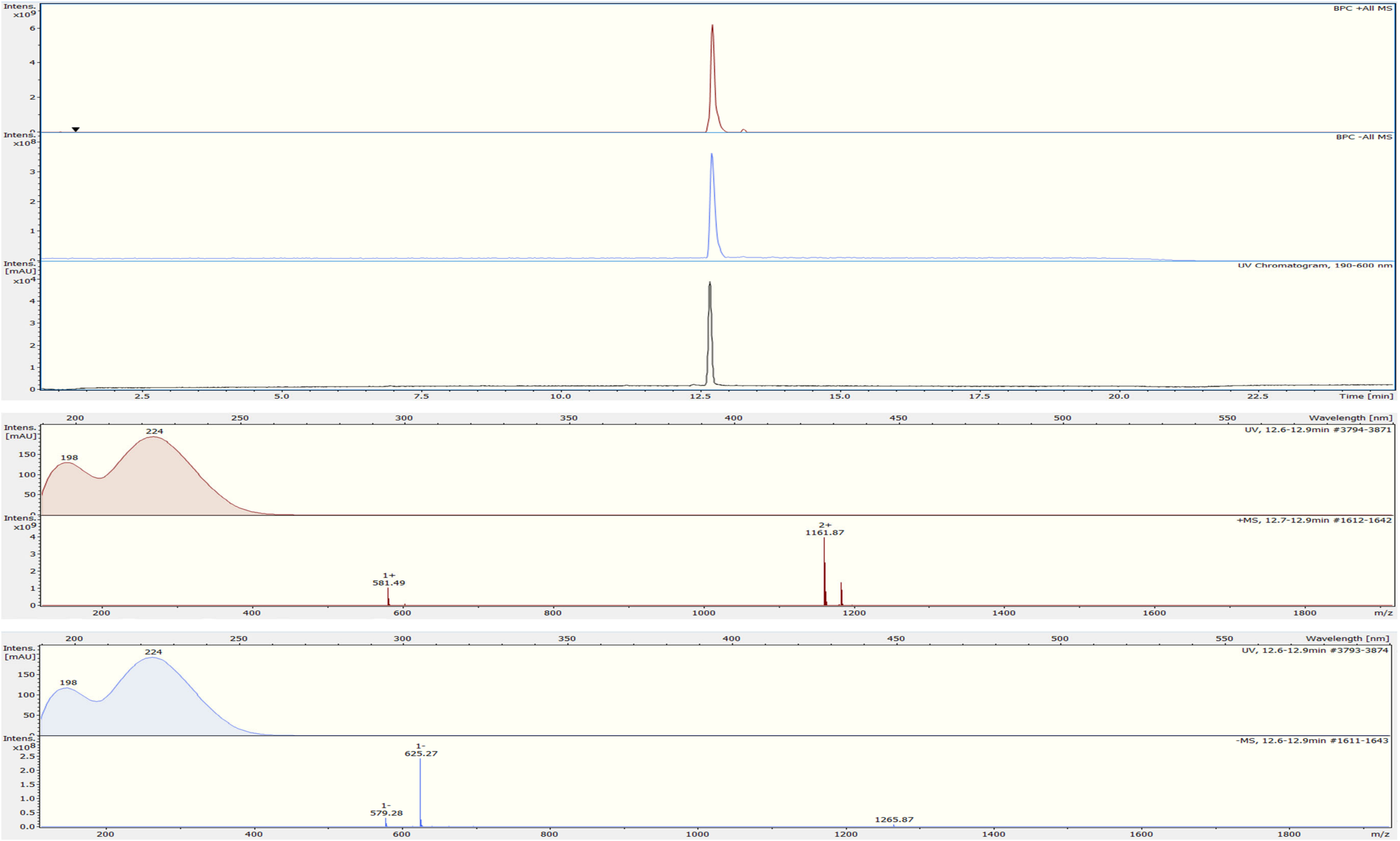

Figure S1. HPLC-ESI-MS spectrum of morinagadepsin (**1**) in positive and negative mode.

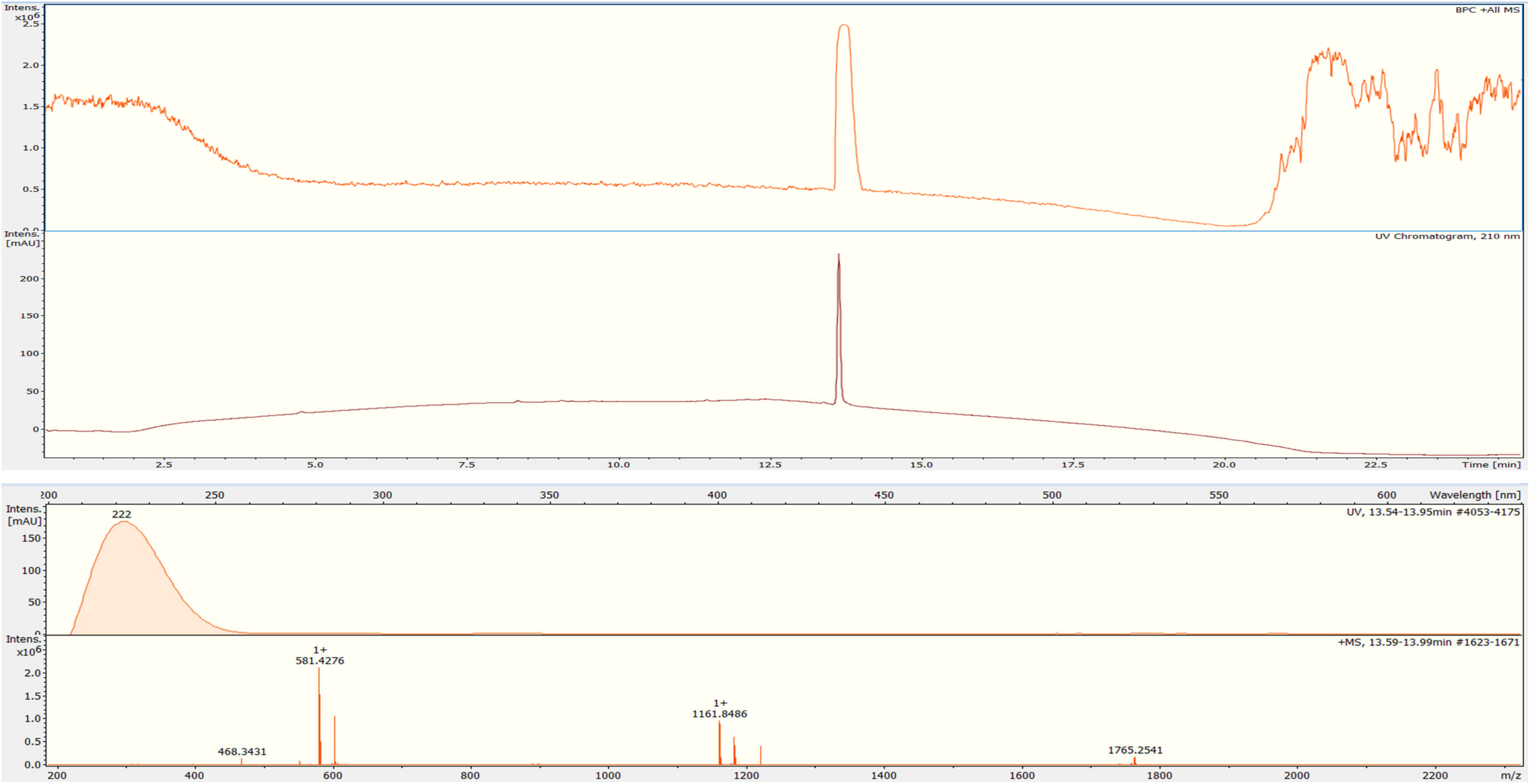

Figure S2. HPLC-HRESI-MS spectrum of morinagadepsin (**1**) in positive mode.

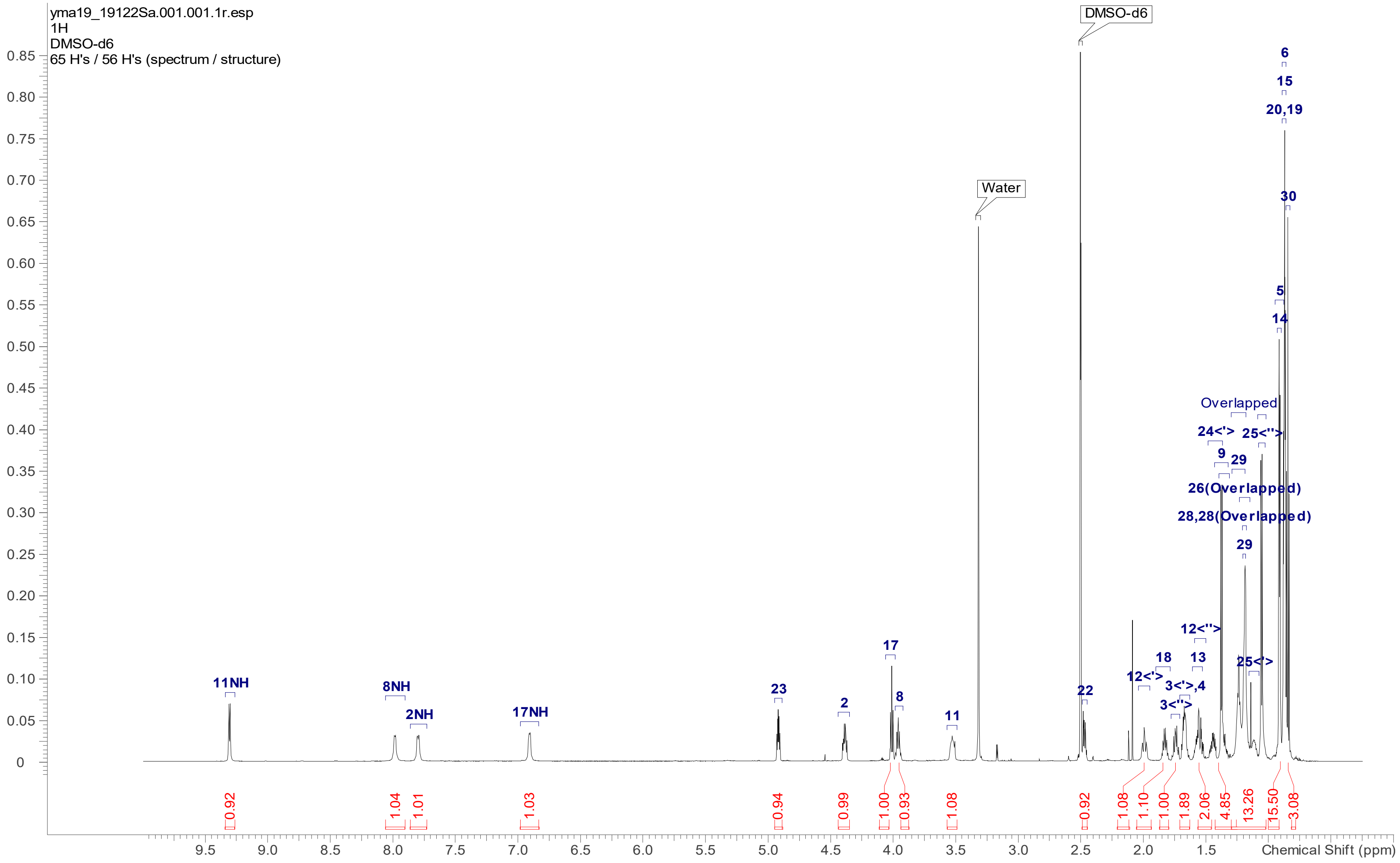

Figure S3. ^1H NMR spectrum (700 MHz, DMSO-d_6) of morinagadepsin (**1**).

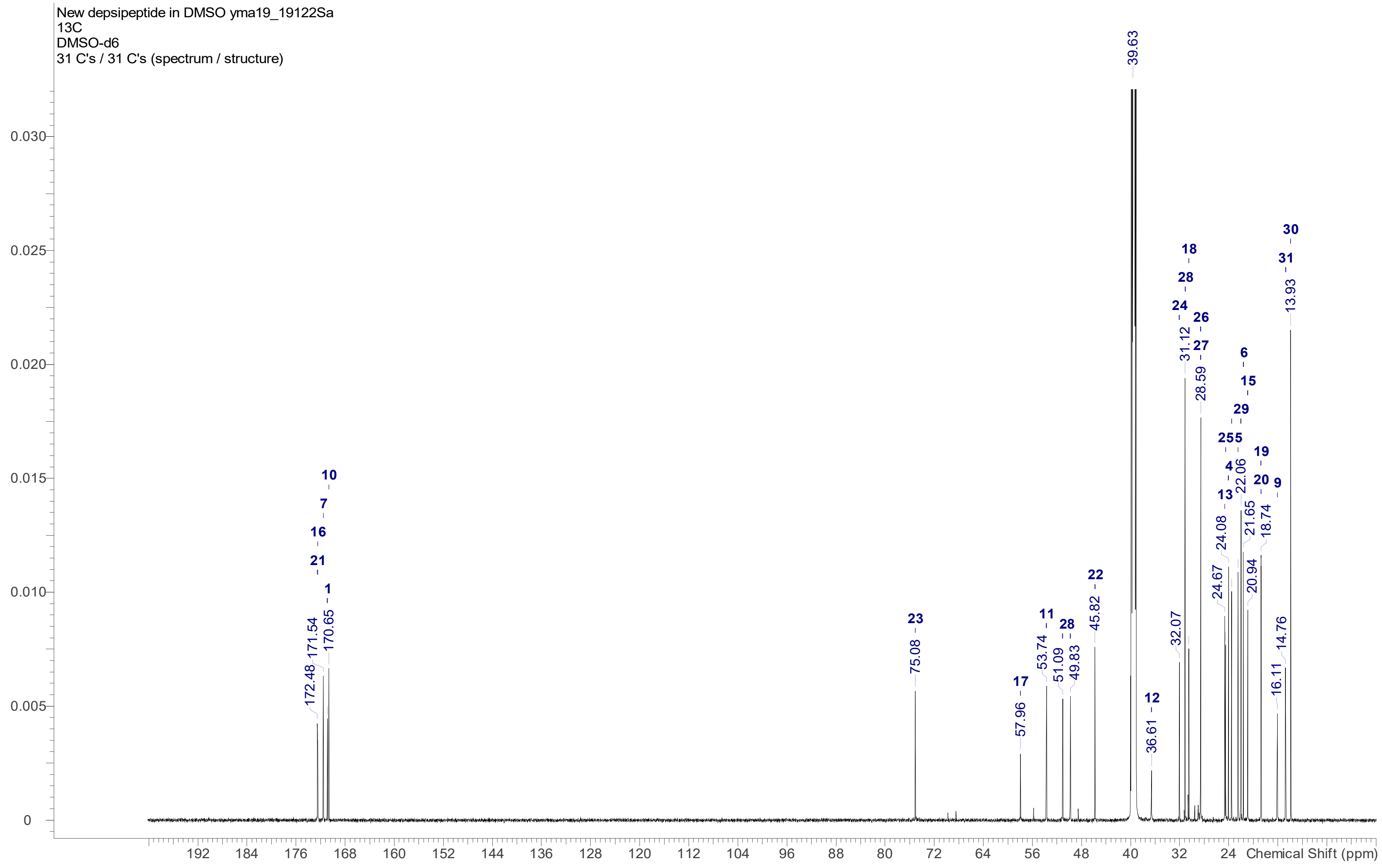

Figure S4. ^{13}C NMR spectrum (175 MHz, DMSO-d_6) of morinagadepsin (**1**).

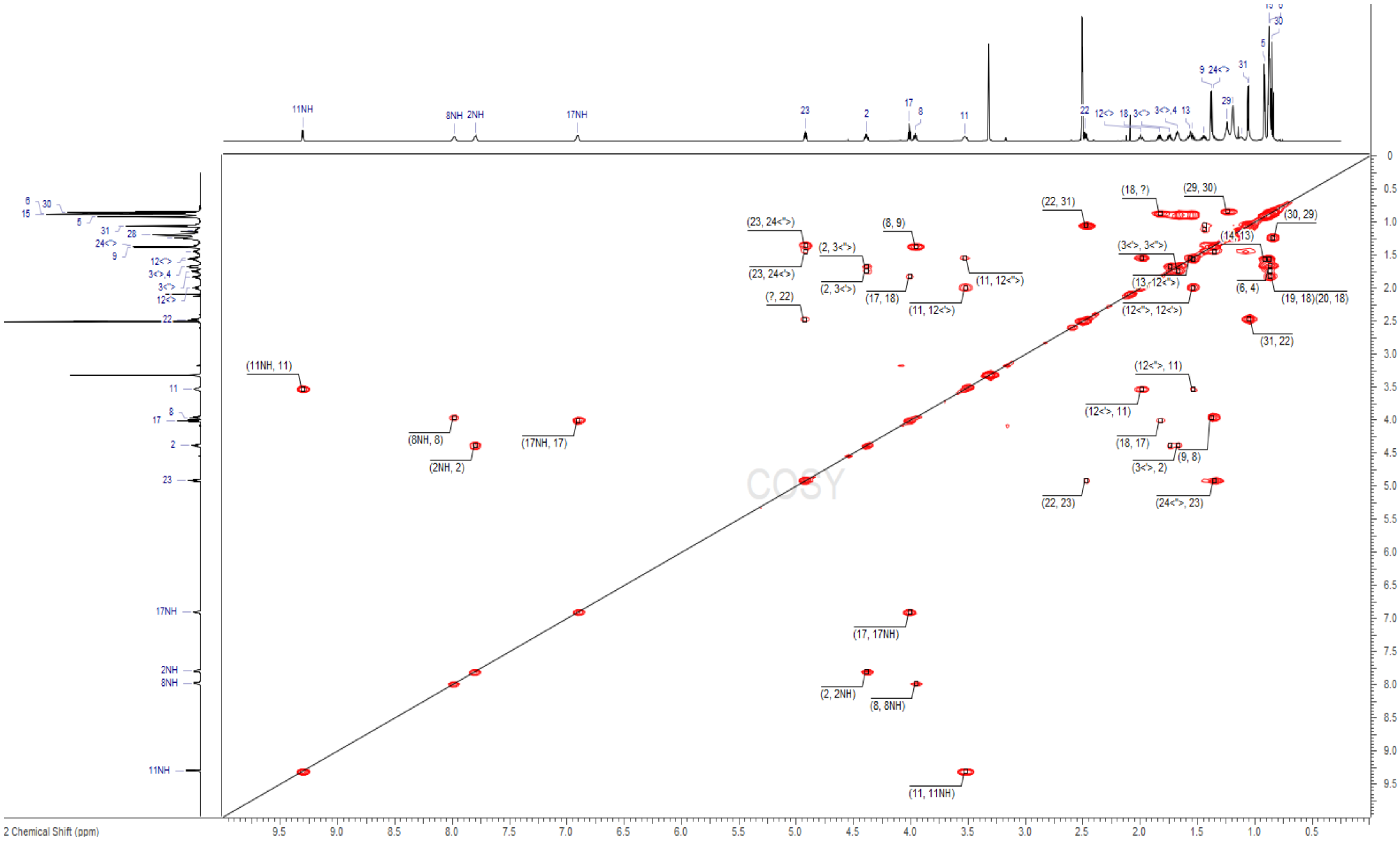

Figure S5. COSY NMR spectrum (700 MHz, DMSO-d_6) of morinagadepsin (**1**).

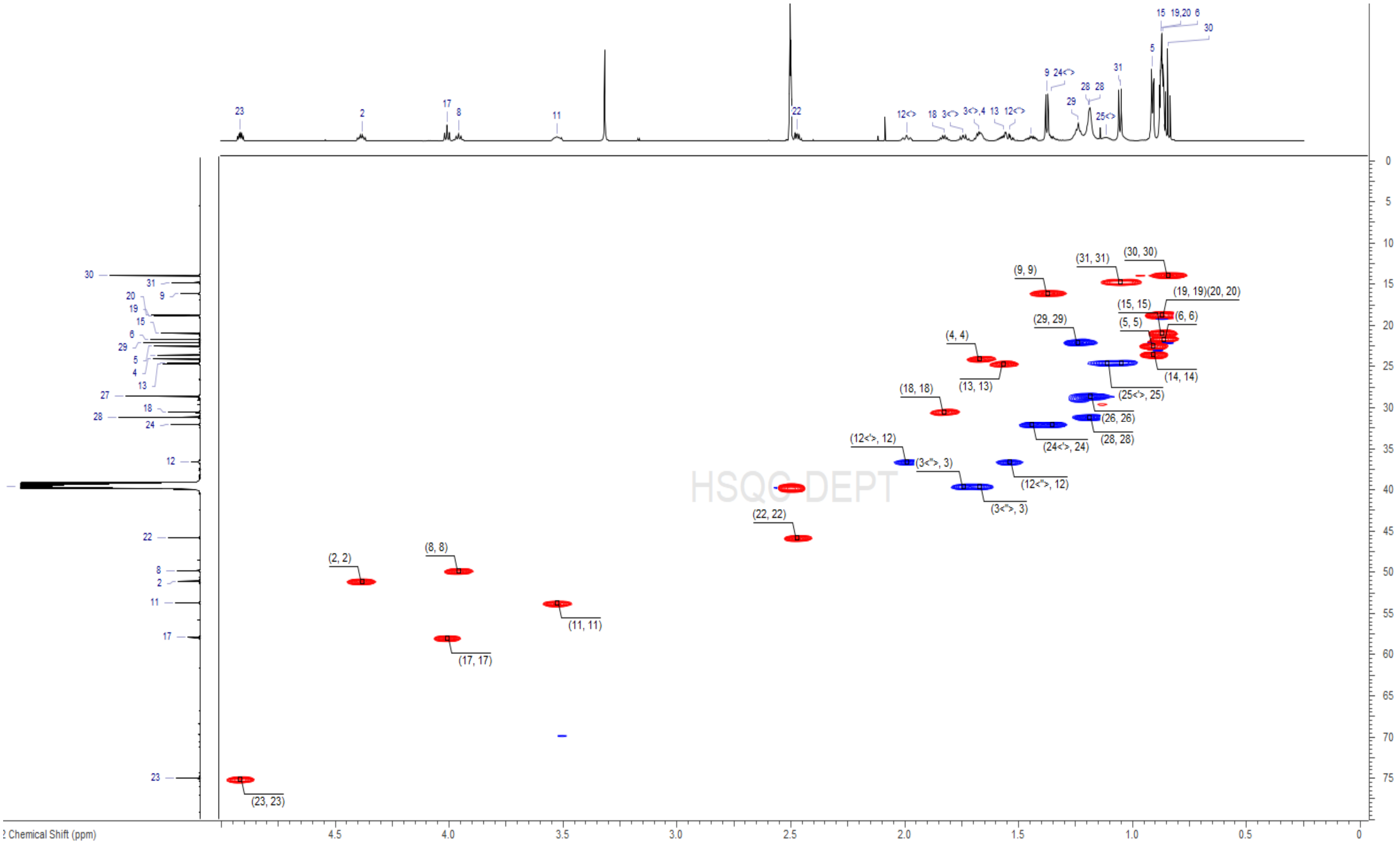

Figure S6. HSQC NMR spectrum (700 MHz, DMSO-d_6) of morinagadepsin (**1**).

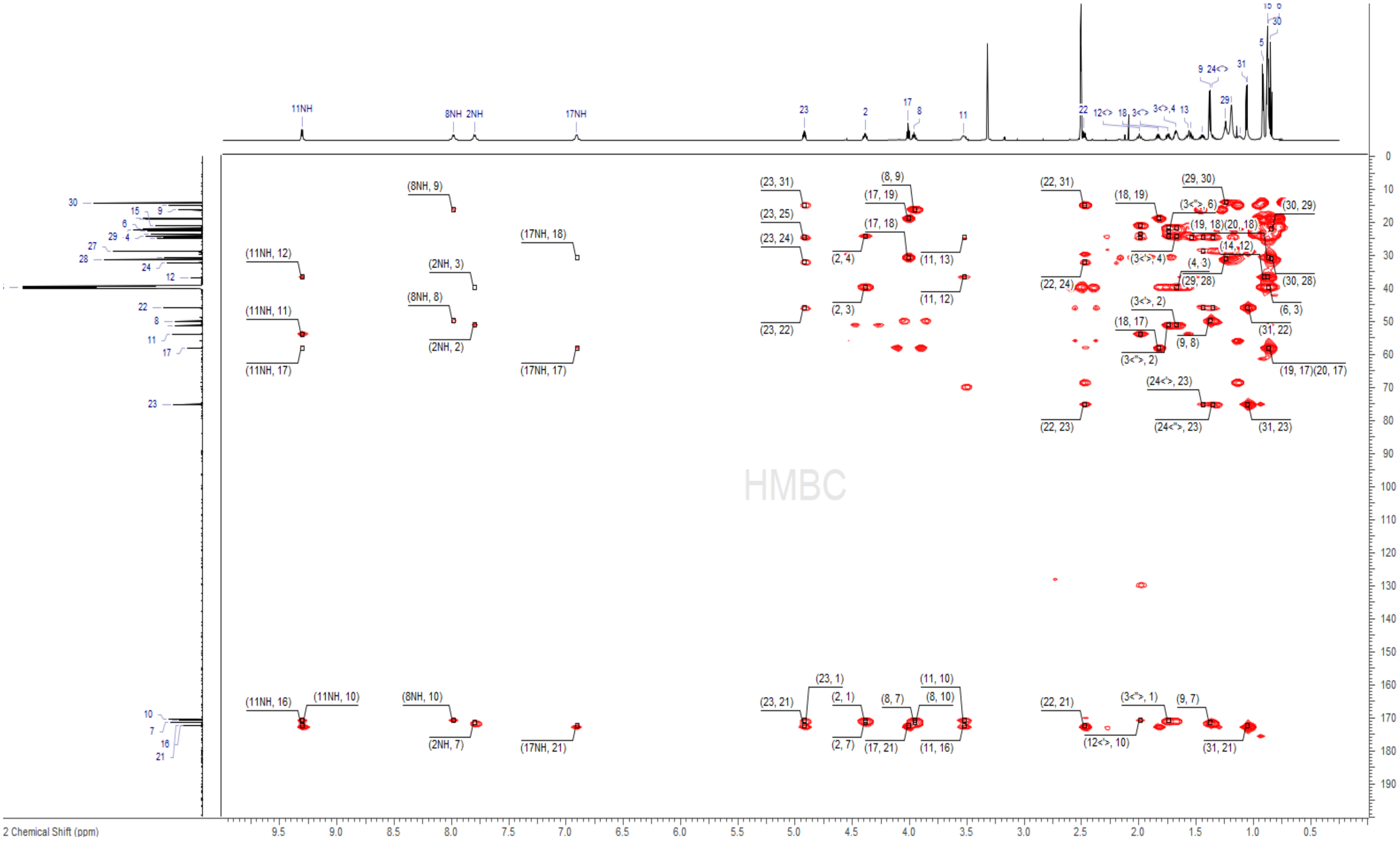

Figure S7. HMBC NMR spectrum (700 MHz, DMSO-d_6) of morinagadepsin (**1**).

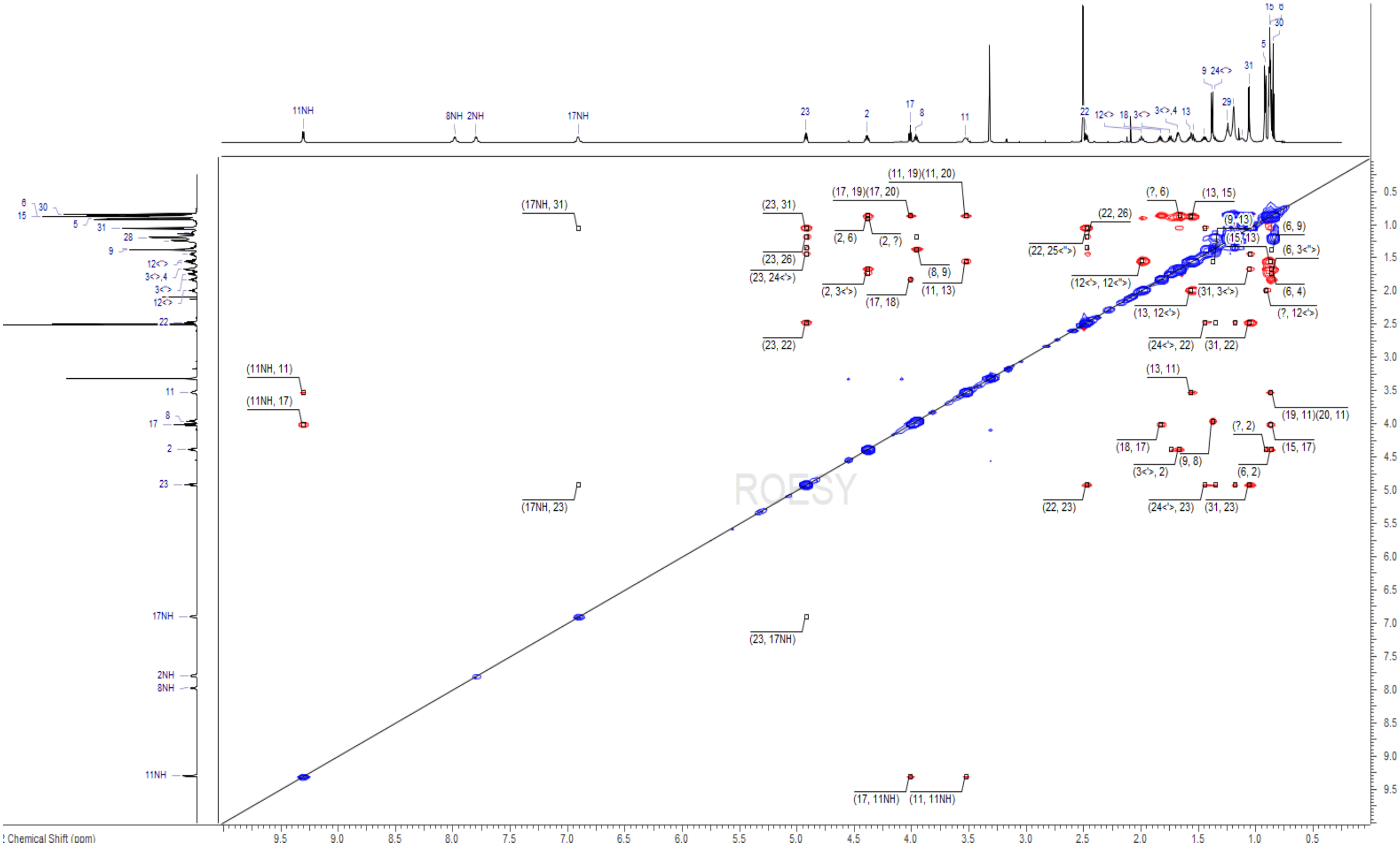

Figure S8. ROESY NMR spectrum (700 MHz, DMSO-d_6) of morinagadepsin (**1**).

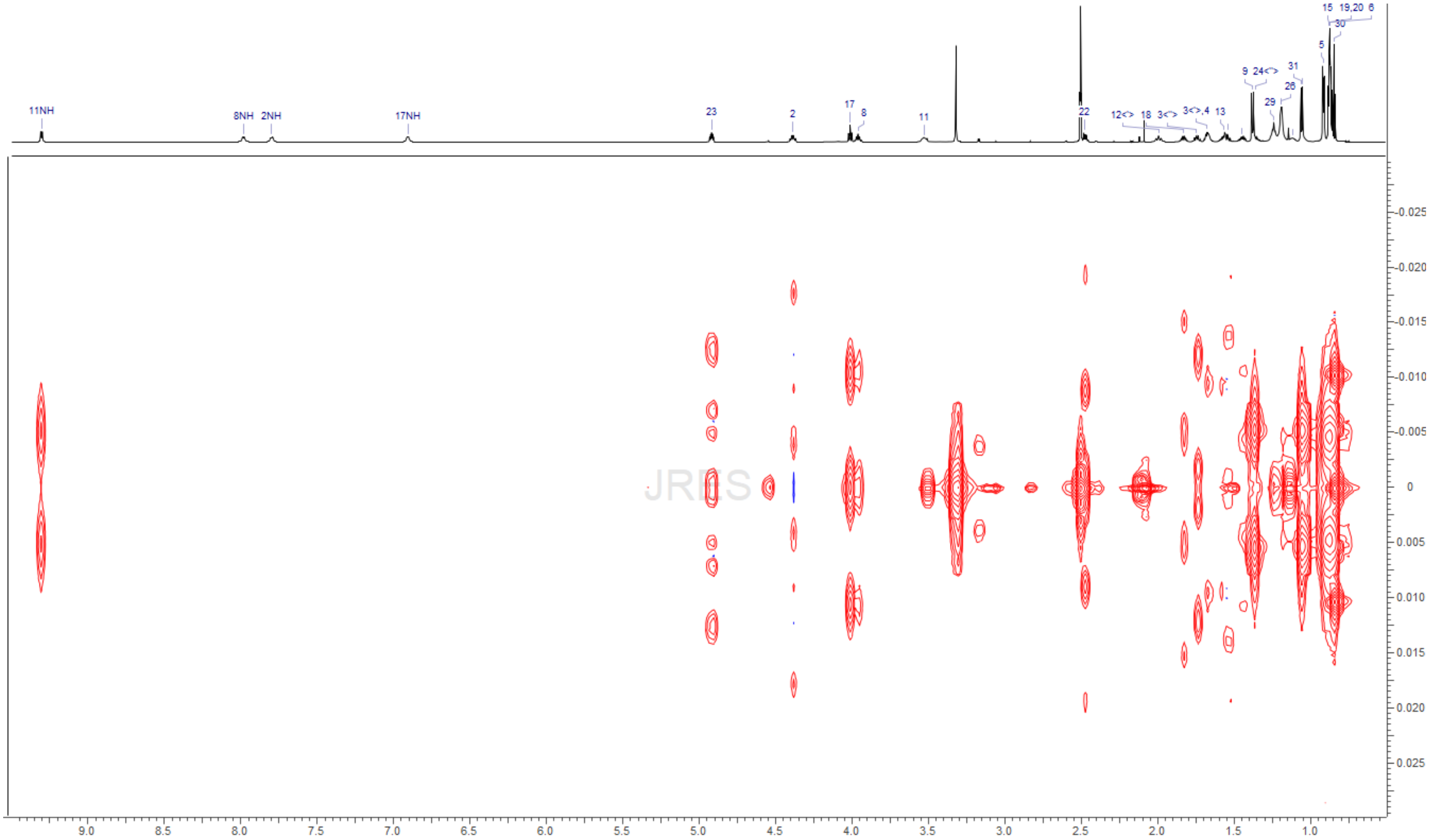

Figure S9. J-res NMR spectrum (700 MHz, DMSO-d_6) of morinagadepsin (**1**).

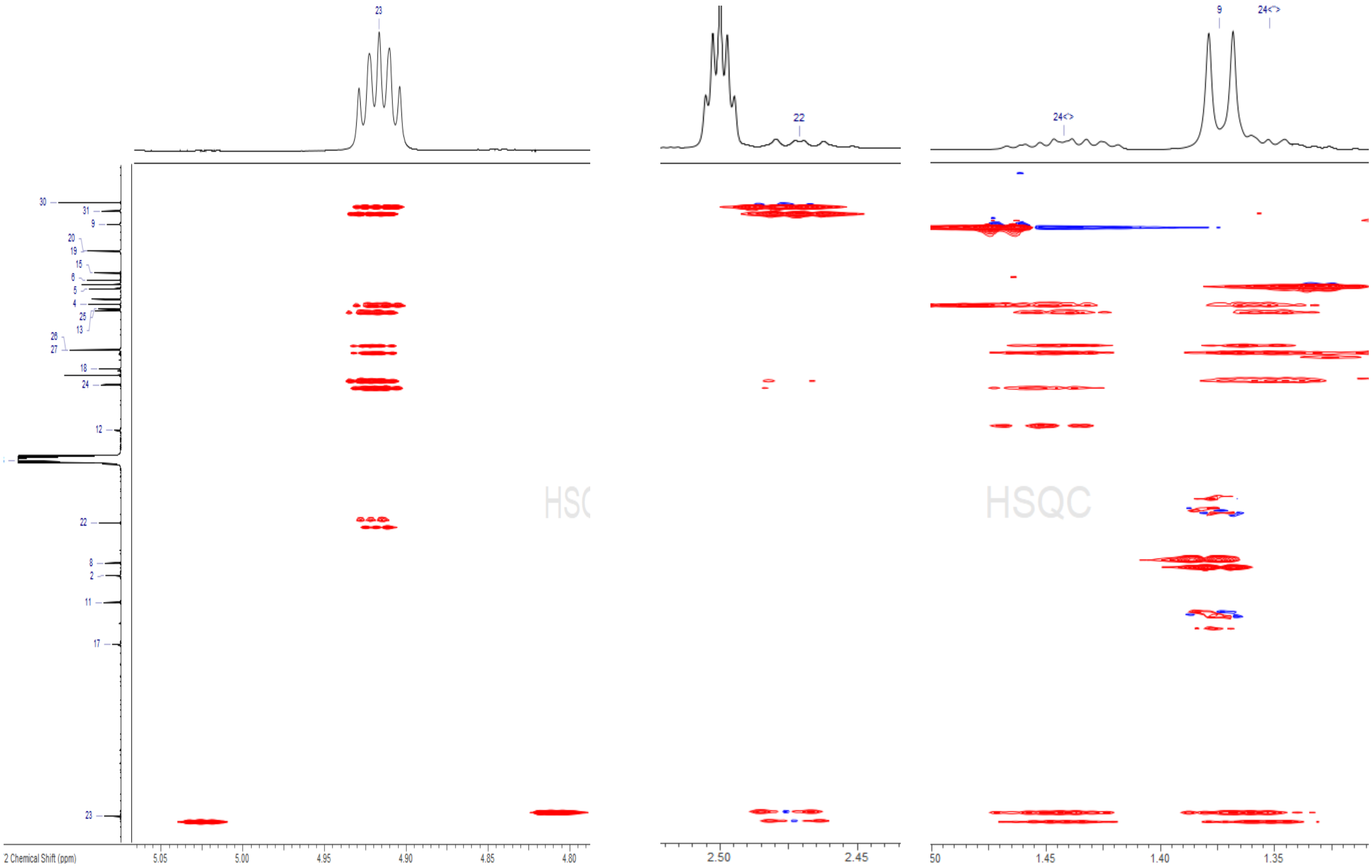

Figure S10. Sections from the HSQC-Hecade NMR spectrum (700 MHz, DMSO-d_6) of morinagadepsin (**1**).

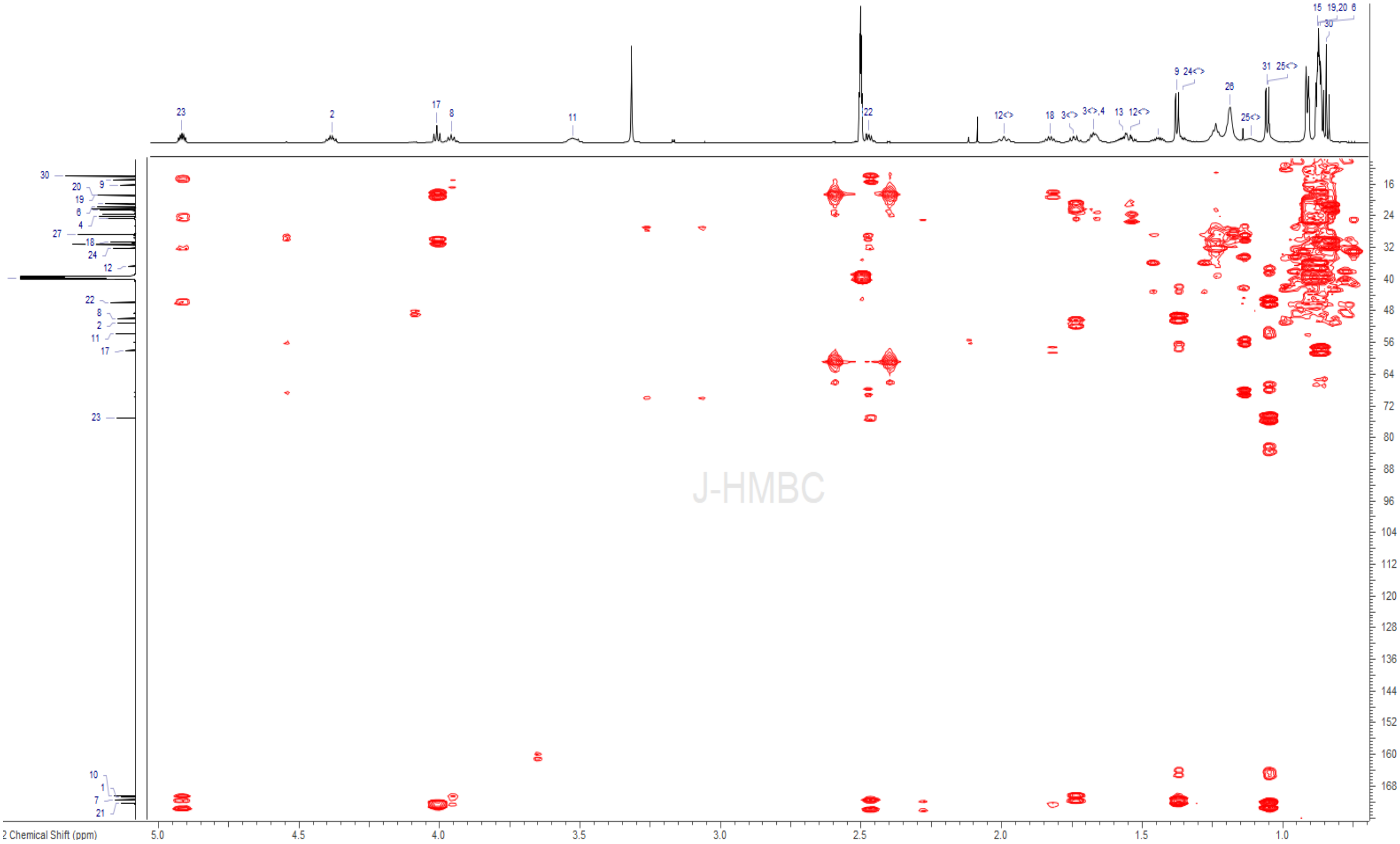

Figure S11. J-HMBC NMR spectrum (700 MHz, DMSO-d_6) of morinagadepsin (**1**).

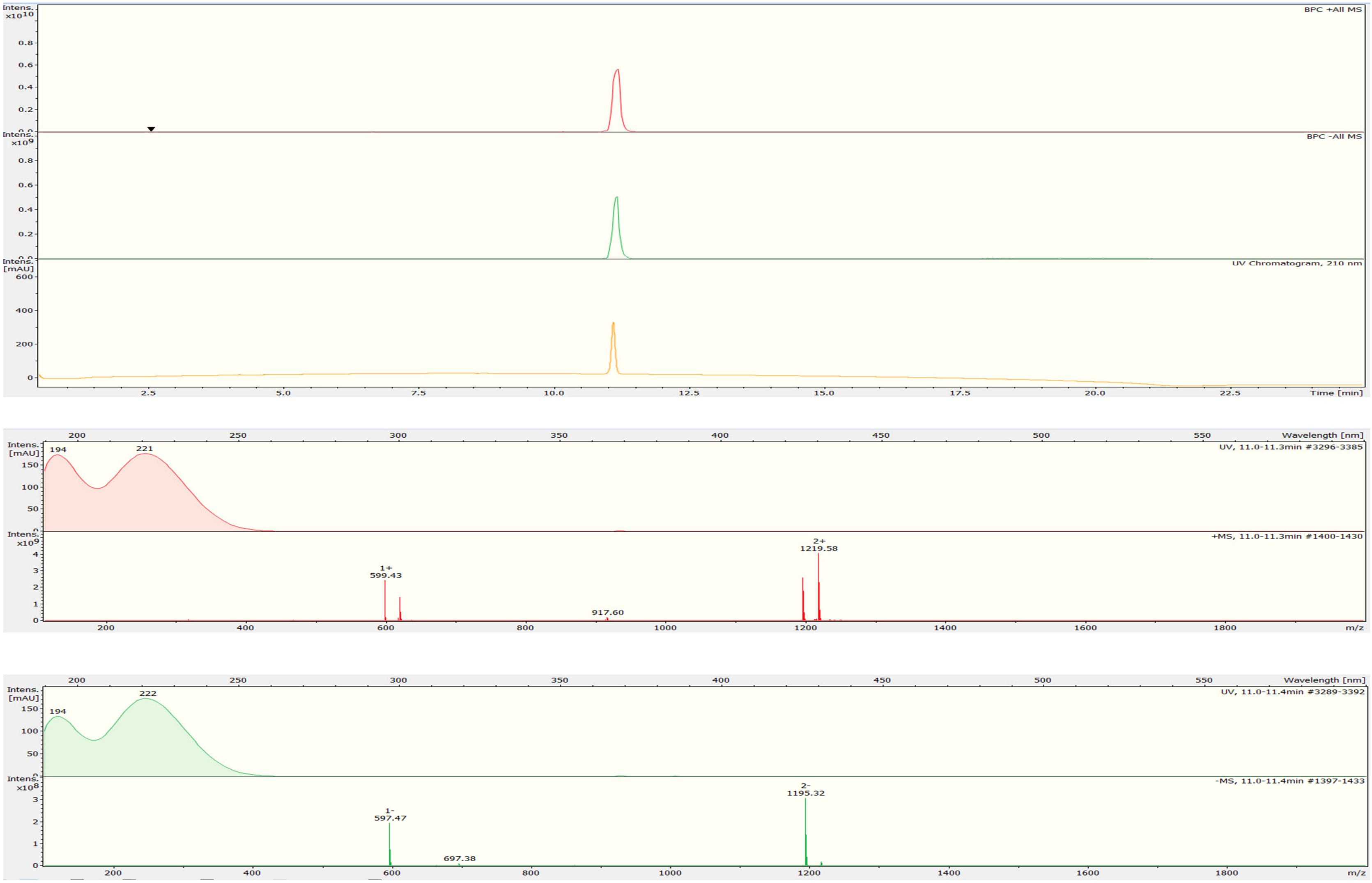

Figure S12. HPLC-ESI-MS spectrum of **2** in positive and negative mode.

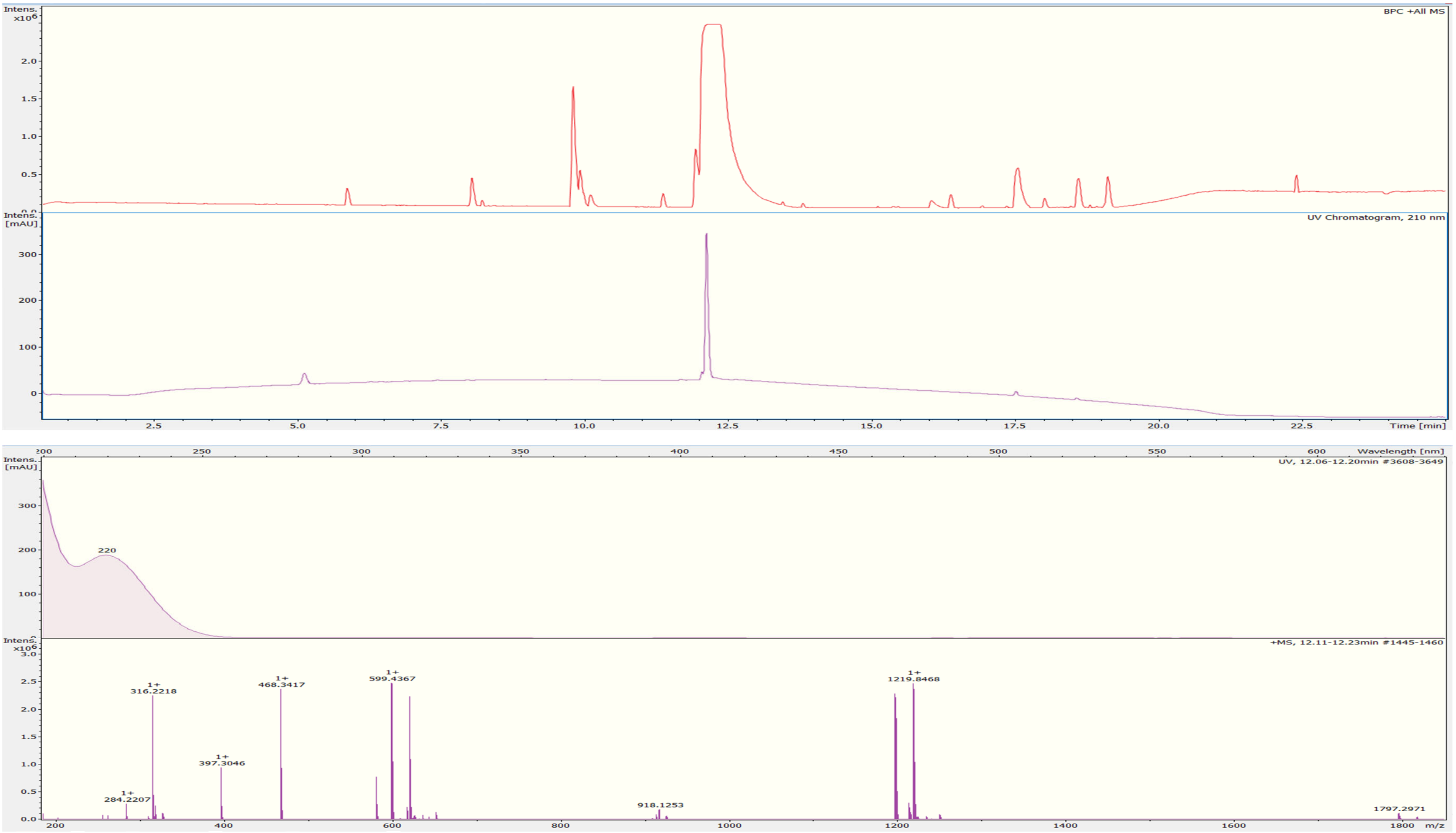

Figure S13. HPLC-HRESI-MS spectrum of **2** in positive mode.

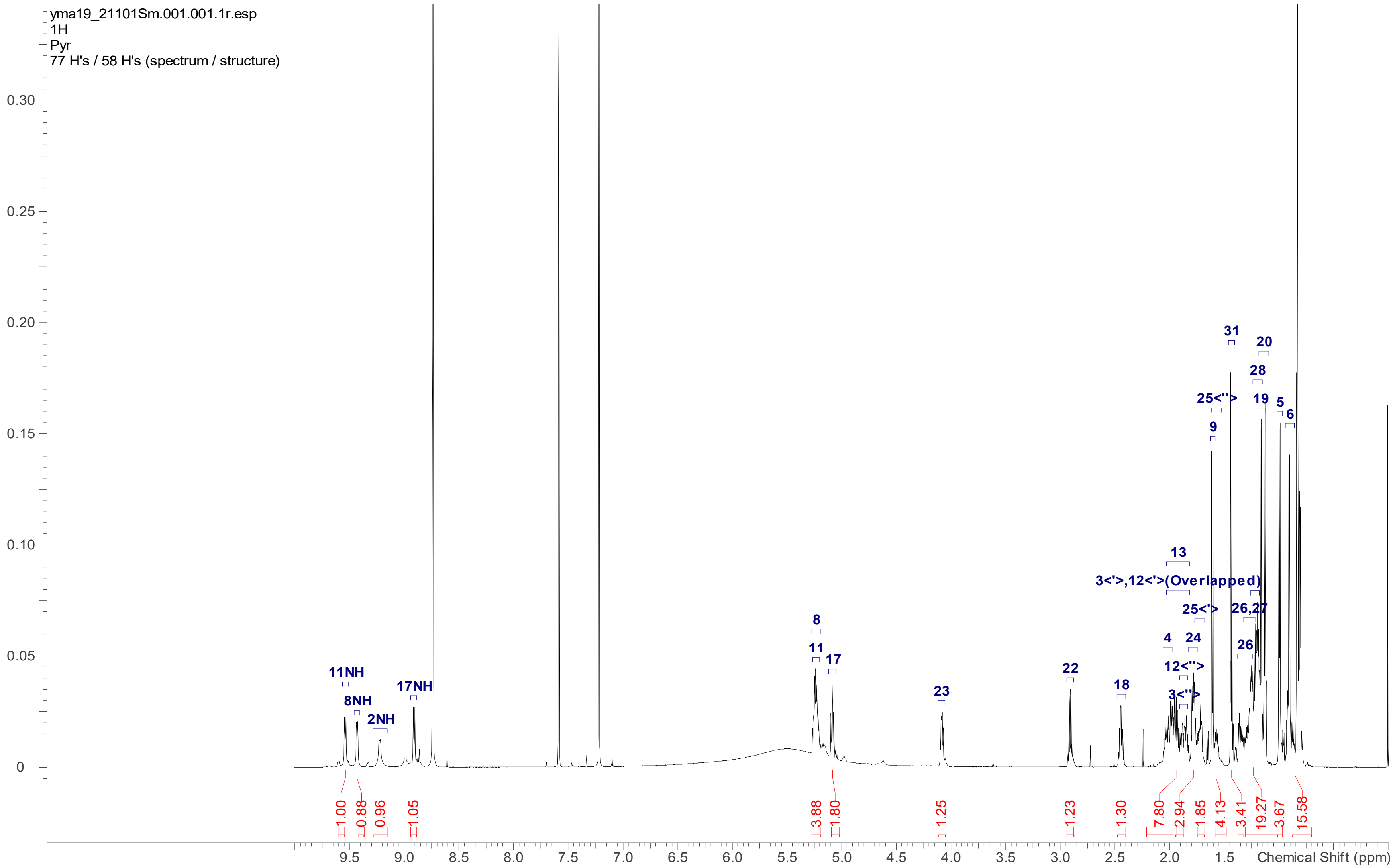

Figure S14. ^{1}H NMR spectrum (700 MHz, pyridine-d_5) of **2**.

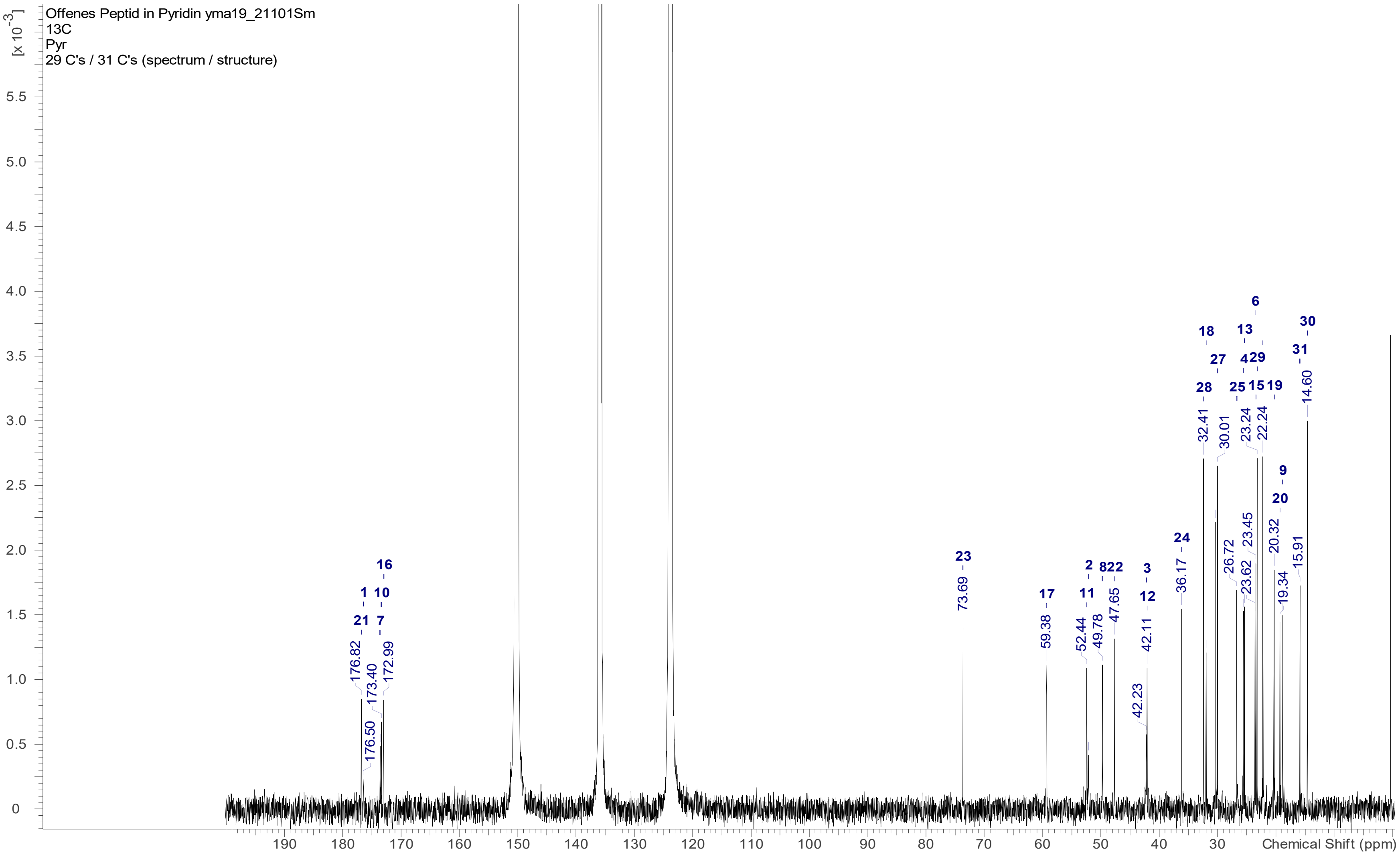

Figure S15. ^{13}C NMR spectrum (175 MHz, pyridine-d_5) of **2**.

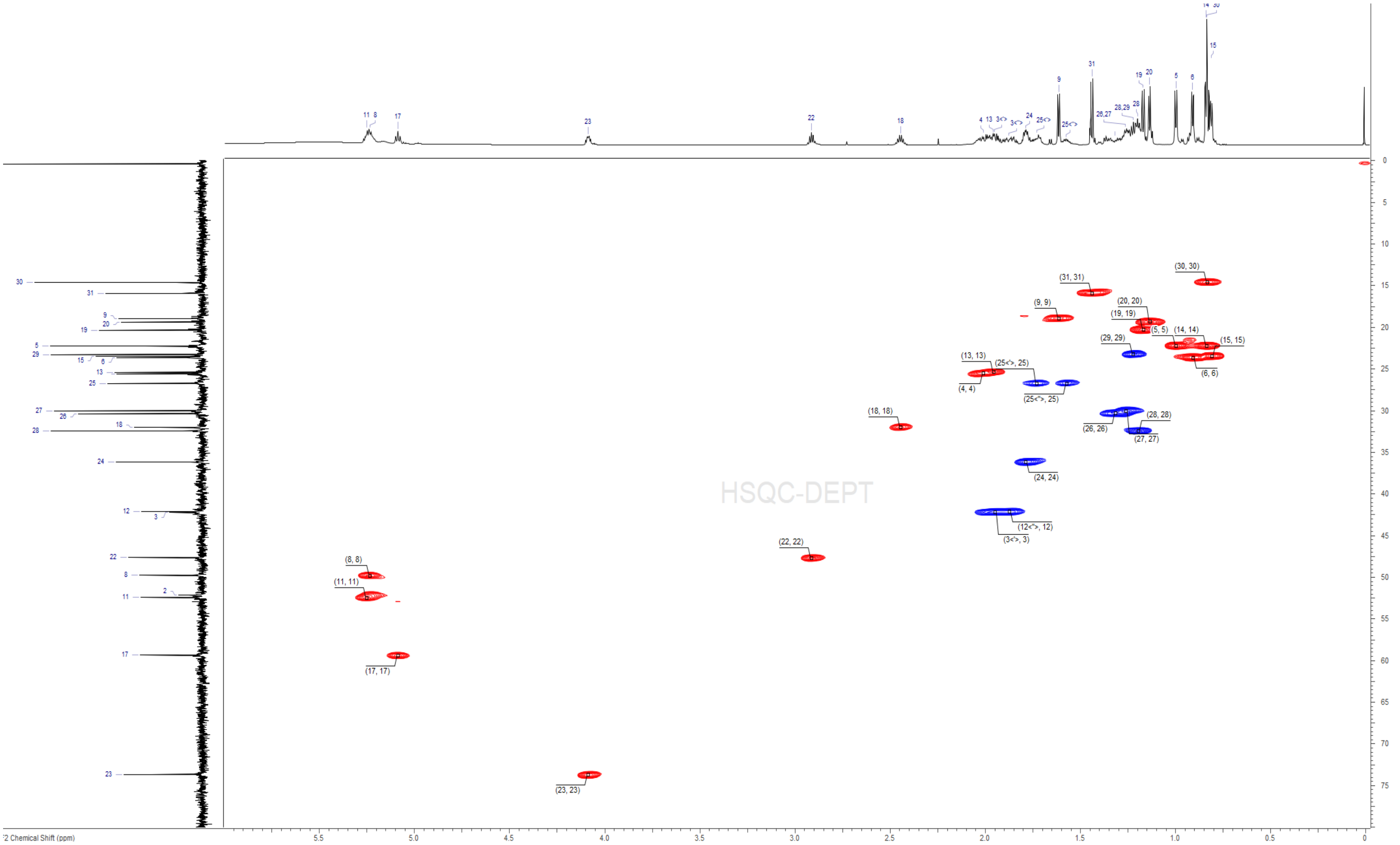

Figure S16. HSQC NMR spectrum (700 MHz, pyridine-d_5) of **2**.

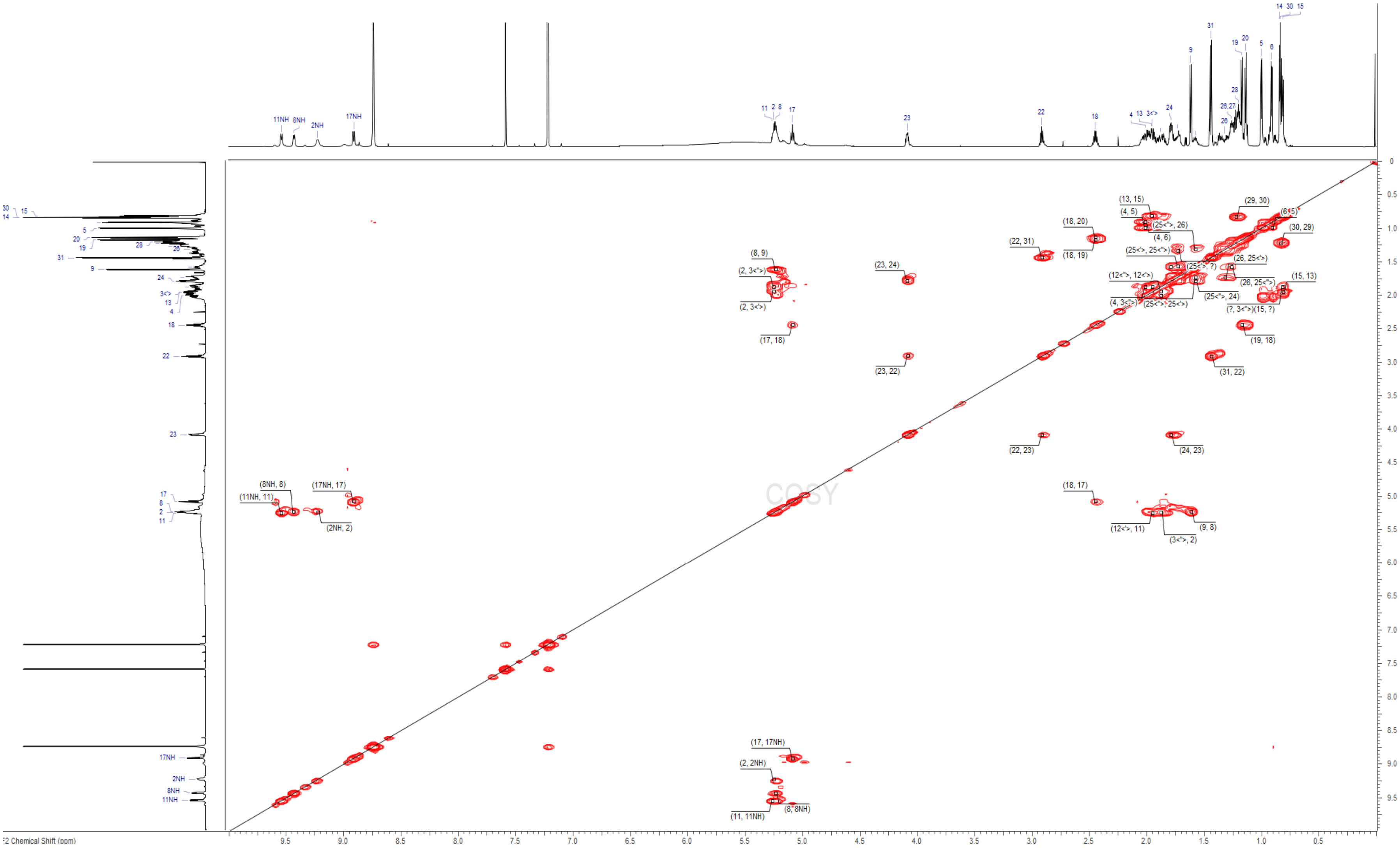

Figure S17. COSY NMR spectrum (700 MHz, pyridine-d_5) of **2**.

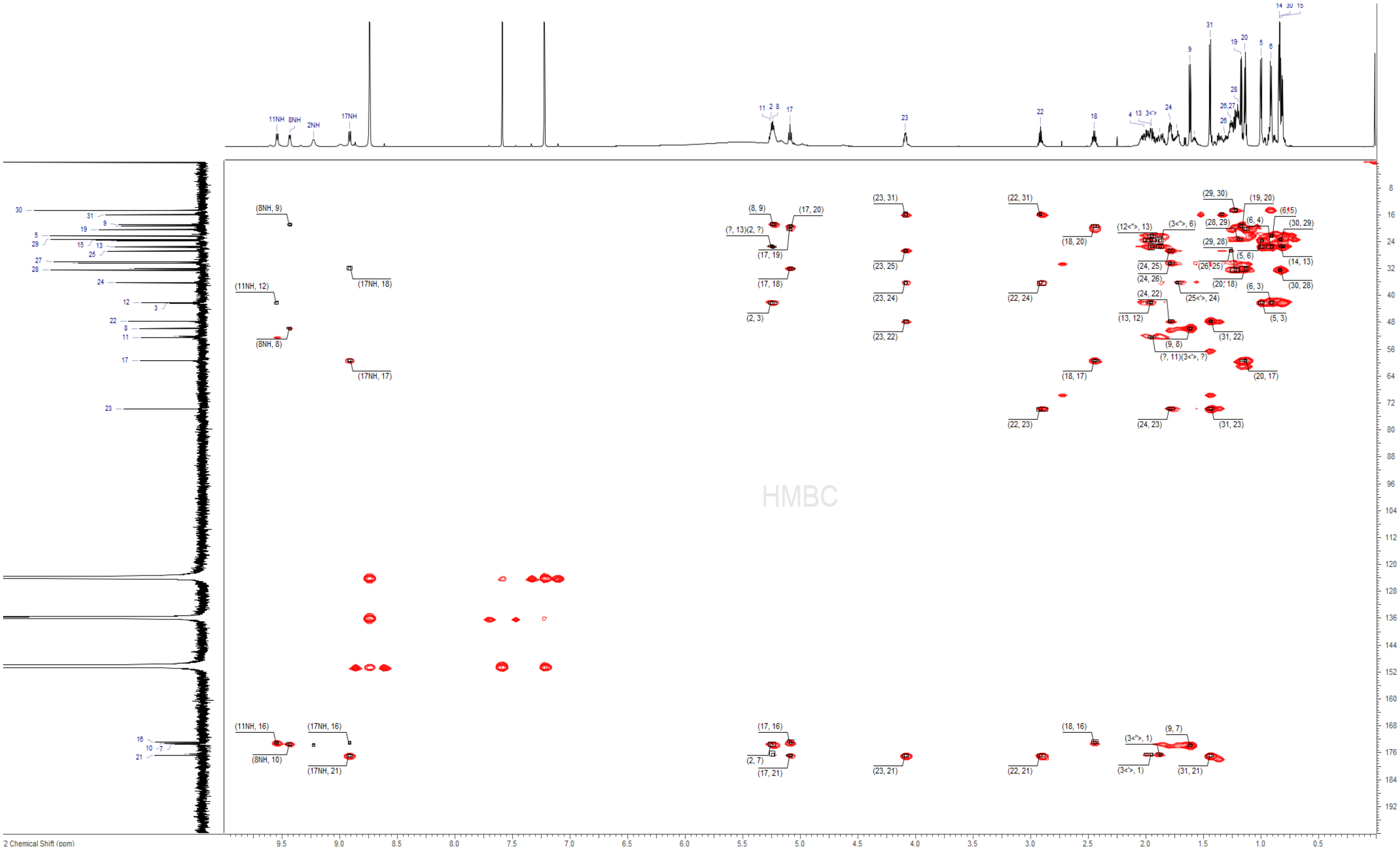

Figure S18. HMBC NMR spectrum (700 MHz, pyridine-d_5) of **2**.

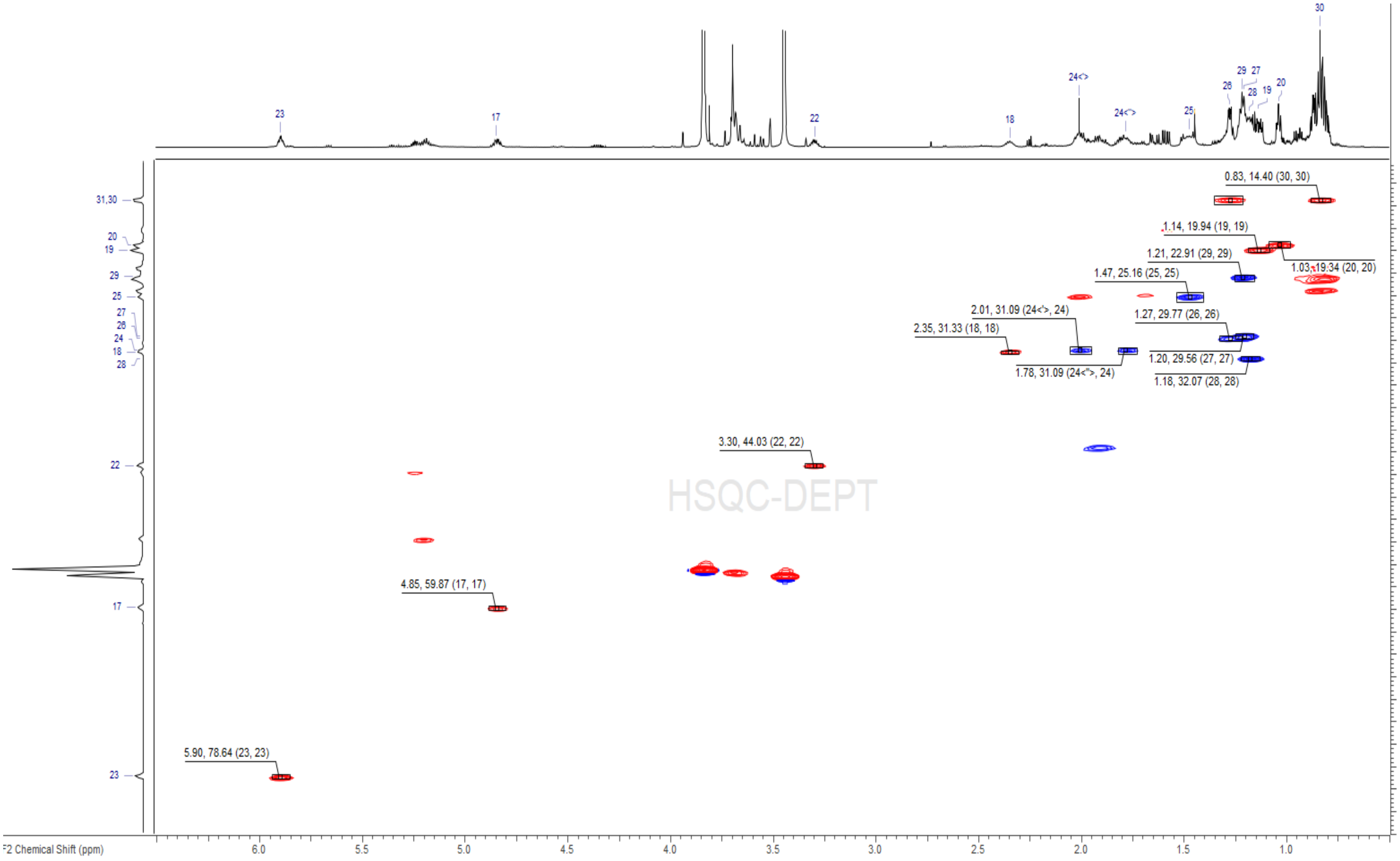

Figure S19. HSQC NMR spectrum (700 MHz, pyridine-d_5) of the S-MTPA-ester of **2**.

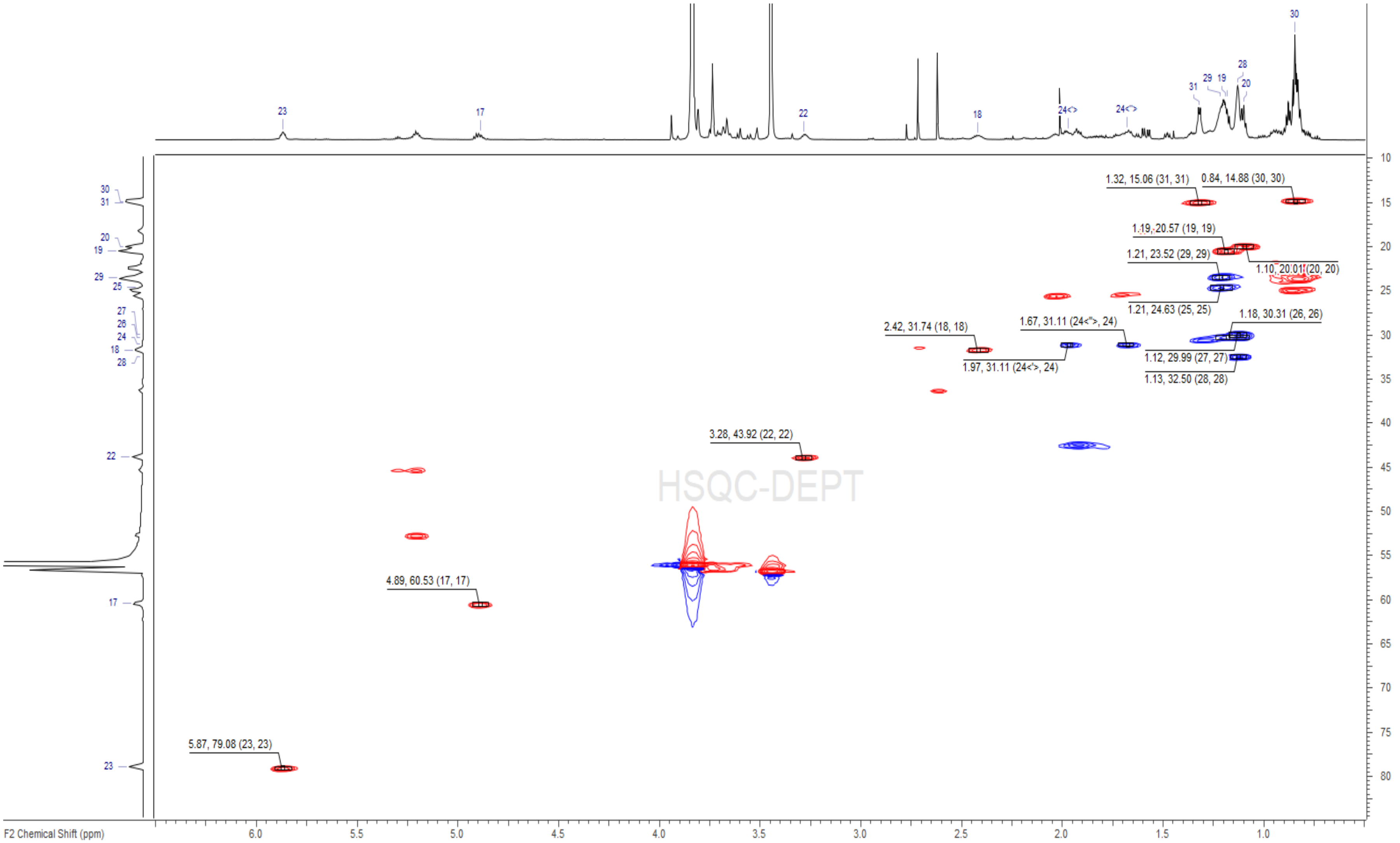

Figure S20. HSQC NMR spectrum (700 MHz, pyridine-d_5) of the R-MTPA-ester of **2**.

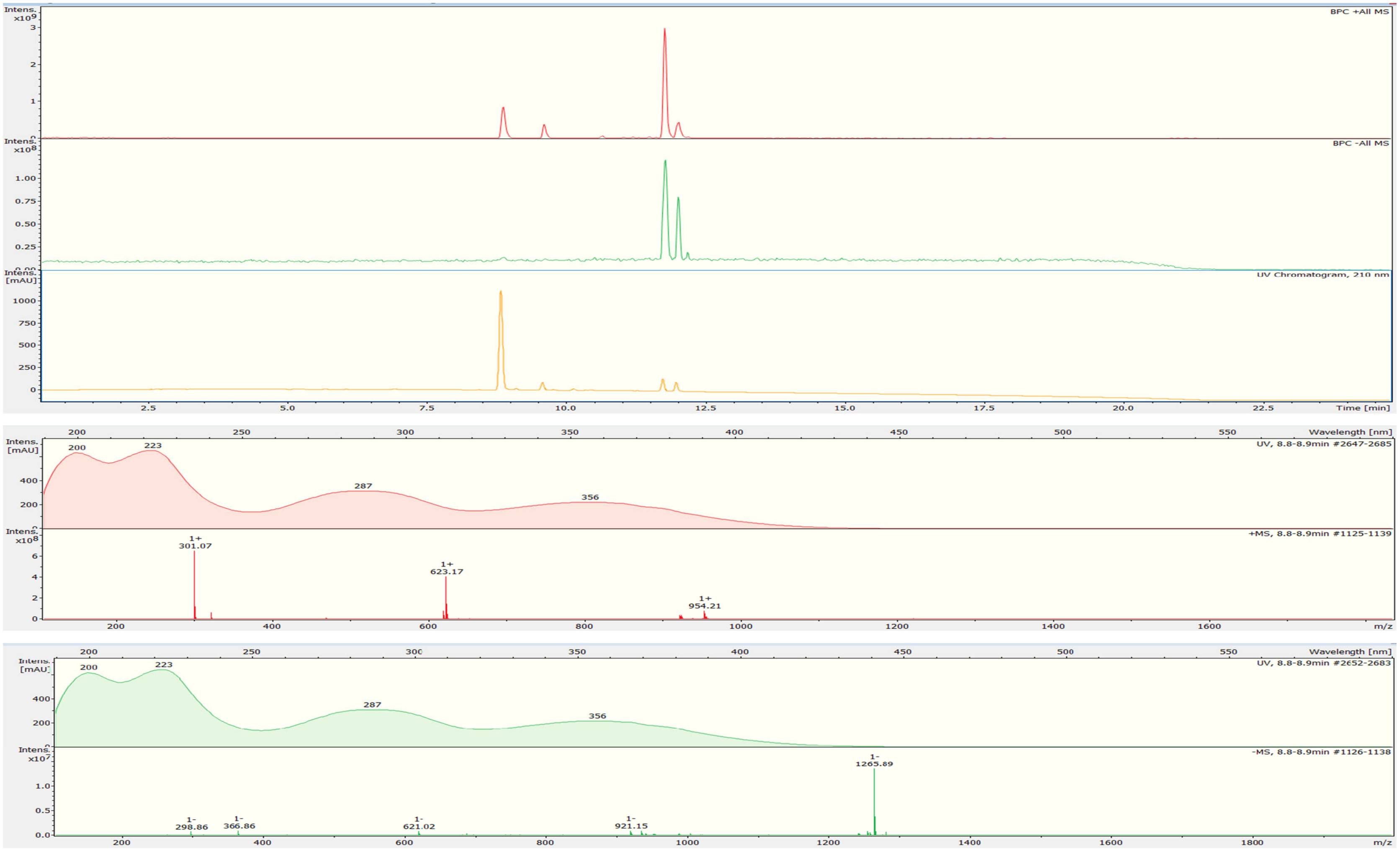

Figure S21. HPLC-ESI-MS spectrum of chaetone B (**3**) in positive and negative mode.

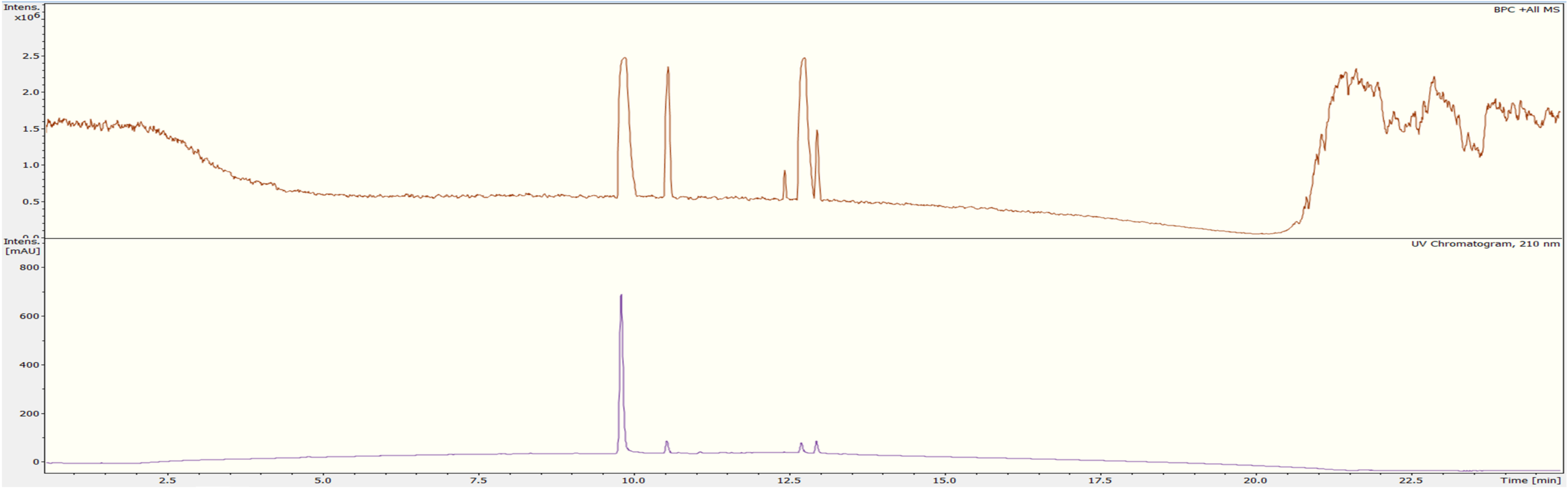

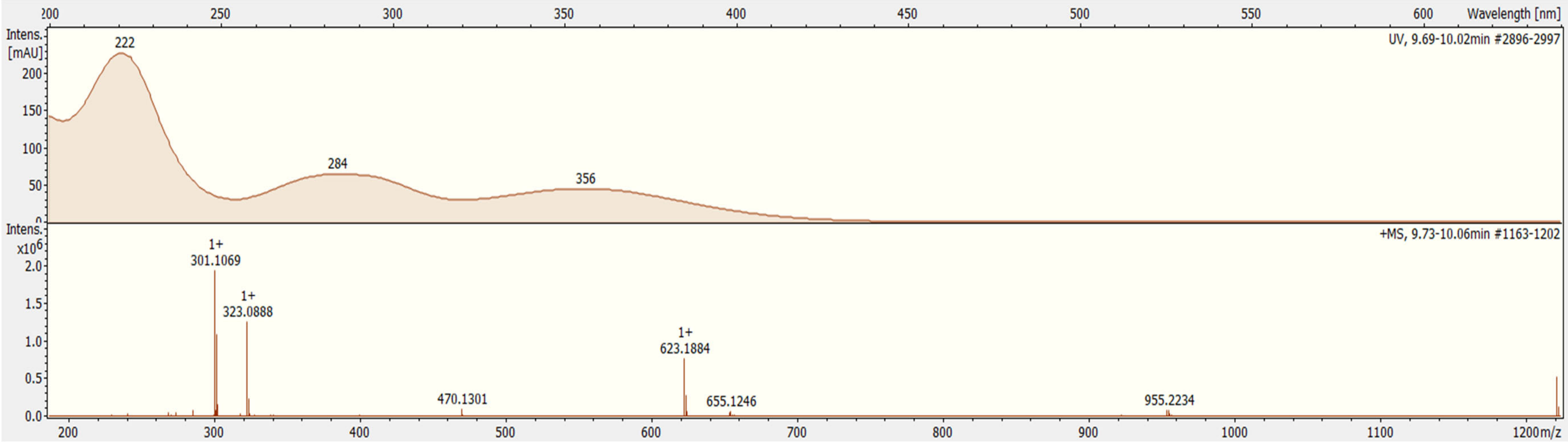

Figure S22. HPLC-HRESI-MS spectrum of chaetone B (**3**) in positive mode.

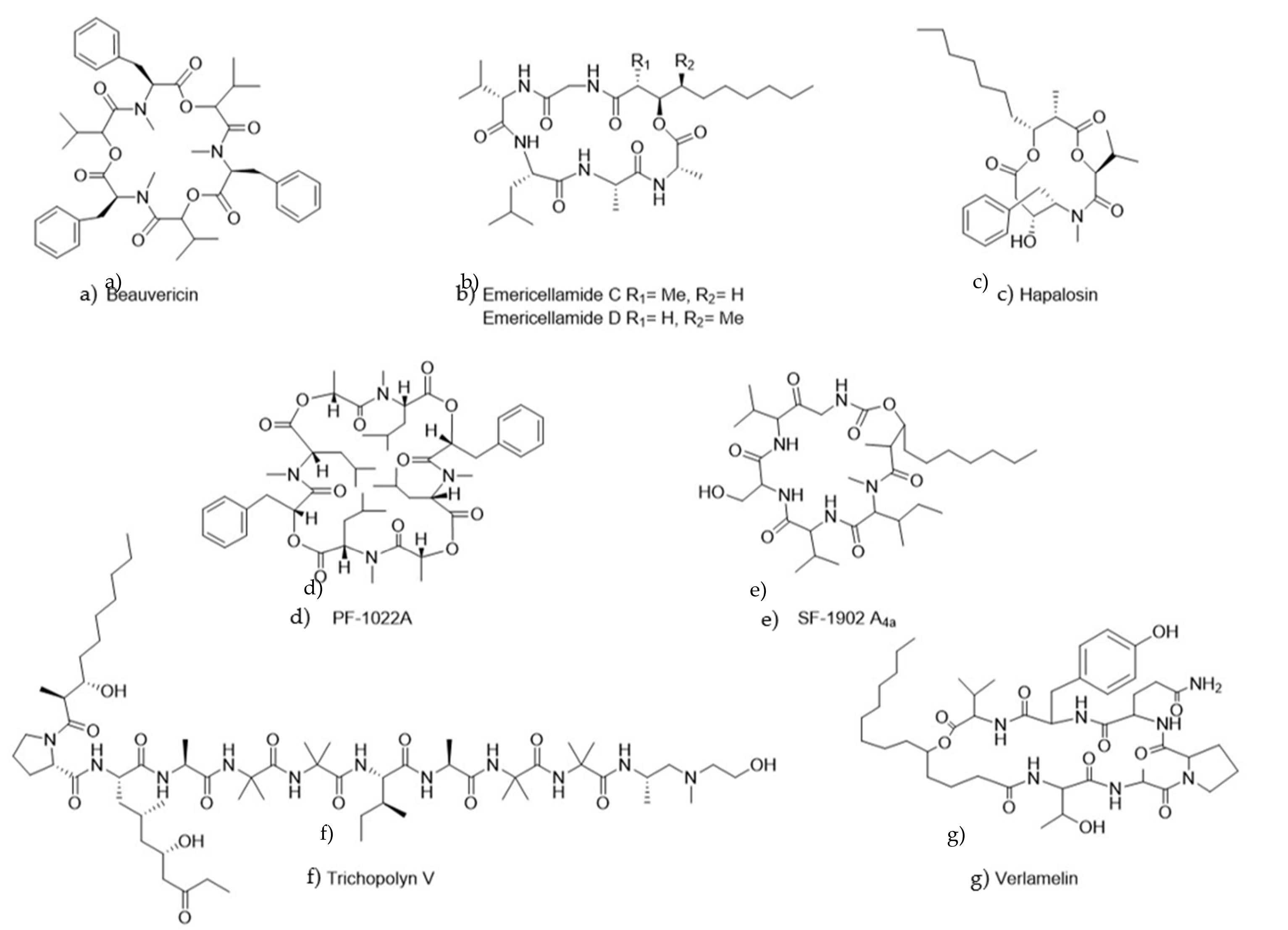

Figure S23. Structures of known depsipeptides.
